# A piRNA-like small RNA interacts with and modulates p-ERM proteins in human somatic cells

**DOI:** 10.1038/ncomms8316

**Published:** 2015-06-22

**Authors:** Yuping Mei, Yuyan Wang, Priti Kumari, Amol Carl Shetty, David Clark, Tyler Gable, Alexander D. MacKerell, Mark Z. Ma, David J. Weber, Austin J. Yang, Martin J. Edelman, Li Mao

**Affiliations:** 1 Department of Oncology and Diagnostic Sciences, University of Maryland School of Dentistry, 650W Baltimore Street, Baltimore, Maryland 21201, USA; 2 Department of Thoracic Medical Oncology, Peking University Cancer Hospital and Institute, Beijing 100142, China; 3 Institute for Genome Sciences, University of Maryland School of Medicine, Baltimore, Maryland 21201, USA; 4 Computer-Aided Drug Design Center, Department of Pharmaceutical Sciences, University of Maryland School of Pharmacy, Baltimore, Maryland 21201, USA; 5 Marlene and Stewart Greenebaum Cancer Center, University of Maryland, 22S Greene Street, Baltimore, Maryland 21201, USA; 6 Department of Biochemistry and Molecular Biology, University of Maryland School of Medicine, Baltimore, Maryland 21201, USA

## Abstract

PIWI-interacting RNAs (piRNAs) are thought to silence transposon and gene expression during development. However, the roles of piRNAs in somatic tissues are largely unknown. Here we report the identification of 555 piRNAs in human lung bronchial epithelial (HBE) and non-small cell lung cancer (NSCLC) cell lines, including 295 that do not exist in databases termed as piRNA-like sncRNAs or piRNA-Ls. Distinctive piRNA/piRNA-L expression patterns are observed between HBE and NSCLC cells. piRNA-like-163 (piR-L-163), the top downregulated piRNA-L in NSCLC cells, binds directly to phosphorylated ERM proteins (p-ERM), which is dependent on the central part of UUNN*UUU*NNUU motif in piR-L-163 and the RRRKPDT element in ERM. The piR-L-163/p-ERM interaction is critical for p-ERM's binding capability to filamentous actin (F-actin) and ERM-binding phosphoprotein 50 (EBP50). Thus, piRNA/piRNA-L may play a regulatory role through direct interaction with proteins in physiological and pathophysiological conditions.

## Abstract (web summary)

PIWI-interacting RNAs (piRNAs) suppress transposon and gene expression during development. Here, the authors identify many piRNAs and piRNA-like small RNAs in 11 human cell lines, and show that one piRNA-like small RNA binds to phosphorylated ERM proteins to regulate cancer cell migration and invasion.

## Graphical abstract

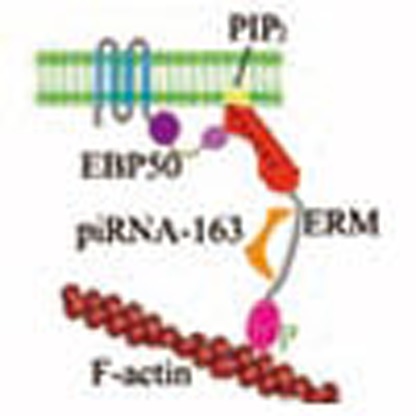

PIWI-interacting RNAs (piRNAs) are the largest class of small noncoding RNAs (sncRNAs) expressed primarily in germline cells and thought to function with PIWI proteins to silence transposon activity and gene expression in a sequence-dependent manner during development^1^. The PIWI-piRNA pathway has been implicated in transposon silencing and repression of gene expression through heterochromatin modification in germline cells^2^,^3^,^4^,^5^,^6^,^7^,^8^,^9^. Although piRNA expression has been observed in human somatic cells such as cancer cells^10^, the extent of piRNA expression in mammalian somatic tissues remains an outstanding question^1^, as are the functional roles of piRNAs in these tissues.

The ERM proteins (ezrin, radixin and moesin) belong to a family of proteins located at cell cortex^11^,^12^,^13^,^14^. They are critical in connecting transmembrane proteins, such as EBP50 (ERM-binding phosphoprotein 50), and the cytoskeleton to play important role in regulating signal transduction pathways^15^,^16^,^17^,^18^,^19^,^20^. These proteins are highly conserved throughout evolution, particularly at their N- and C-terminus regions^21^,^22^,^23^,^24^,^25^, and expressed in a tissue-specific manner^11^,^26^. It is believed that ERM function is regulated through changing the folding of the protein^12^,^13^,^14^,^22^. In the inactive form, the proteins are folded and the binding sites to EBP50 and filamentous actin (F-actin) are masked^22^,^23^. Upon phosphorylation at a particular C terminus site (Thr558 for moesin or Thr576, and Thr564 for ezrin and radixin, respectively), ERM proteins become activated by unfolding to expose the masked binding sites and allowing the proteins bind to EBP50 and F-actin^12^,^13^,^14^,^17^,^18^,^19^.

Here we demonstrated that piRNAs are expressed in somatic human bronchial epithelial (HBE) cells, and the expression patterns are distinctive between normal bronchial epithelial cells and lung cancer cells. Importantly, we further demonstrated that piRNA-like-163 (piR-L-163), the top downregulated piRNAs in lung cancer cells, directly binds to phosphorylated ERM (p-ERM) and play a critical role in ERM activation.

## Results

### Expression of piRNAs/piRNA-like sncRNAs in somatic cells

**Figure 1:**
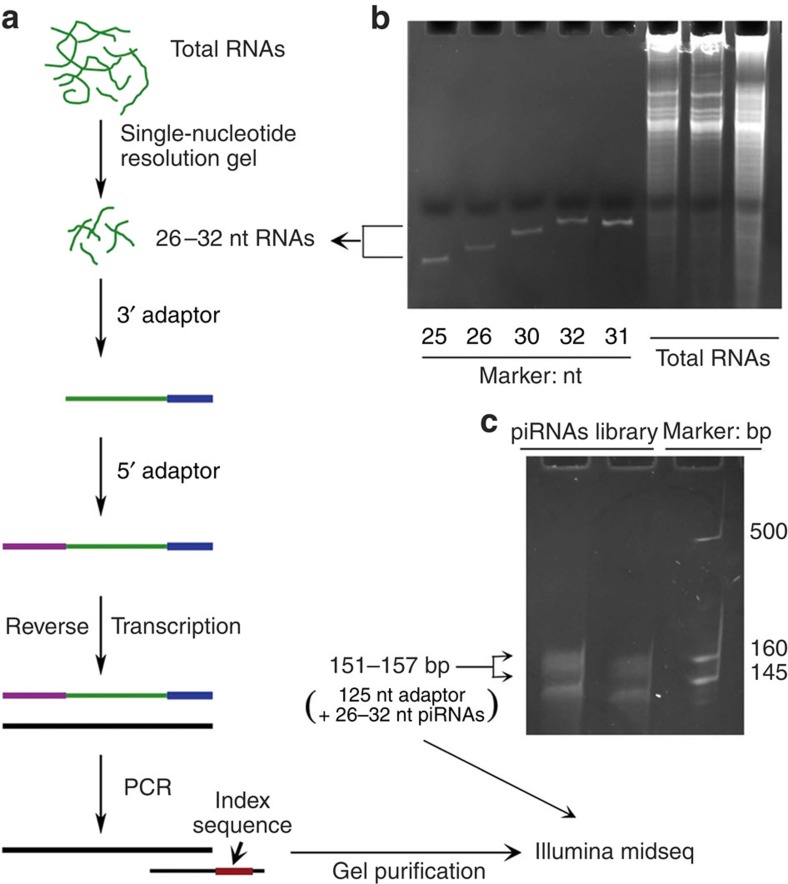
Flowchart of the protocol used to prepare sncRNAs from the cell lines for RNA sequencing. (**a**) Outlined steps of the preparation. (**b**) Size-guided sncRNA extraction at 1 nt resolution. (**c**) Secondary sncRNA purification after library construction based on sizes for RNA sequencing. The smaller sized products (low band) are likely microRNAs.

To explore potential implications of piRNA in lung cancer, we first analysed global piRNA expression profiles in eight non-small cell lung cancer (NSCLC) and three HBE cell lines. Small RNAs ranging approximately from 25 to 33 bases were used for library construction (Fig. 1a–c and Table 1) based on previous reports^27^,^28^,^29^,^30^,^31^,^32^. RNA sequencing was performed and resulted in ∼4.5 million reads with >99% of the reads between 26 and 32 bases (Supplementary Fig. 1a and Supplementary Table 1), and ∼50% of the reads mapped to ≥2 loci in the human genome sequences (Supplementary Fig. 1b), indicating that the piRNA reads captured a nontrivial portion of piRNA diversity^33^.

**Figure 2:**
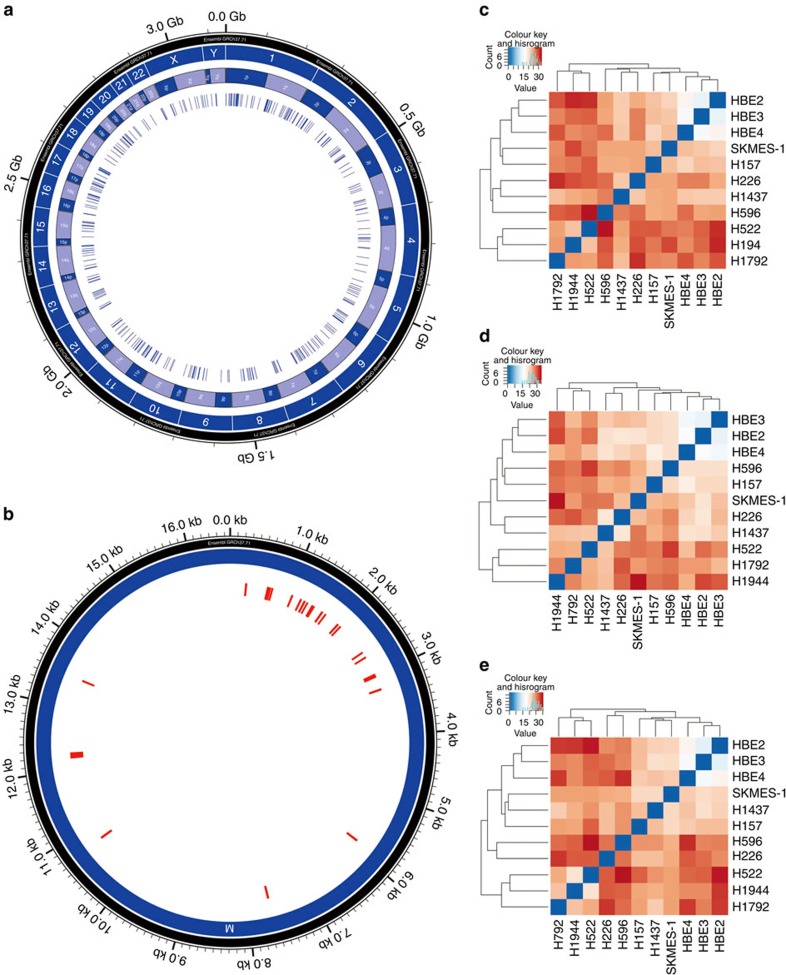
Genome distribution and clustering analysis of piRNAs and piRNA-Ls expressed in HBE and NSCLC cells. (**a**) Distribution of piRNAs and piRNA-Ls in chromosomes. (**b**) Distribution of piRNAs and piRNA-Ls in mitochondria genome. Clustering based on piRNA and piRNA-L expression patterns in HBE2-4, ADC (SK-MES-1, H157, H226 and H1437) and SCC (H596, H522, H194 and H1792). (**c**) Entire piRNA and piRNA-L expressed, (**d**) piRNAs only and (**e**) piRNA-Ls only.

A total of 555 piRNAs between 26 and 32 bases were called based on ≥20 reads of individual piRNA in any of the cell lines. These piRNAs are distributed among chromosomal and mitochondrial genomes with bias in chromosomes 1 and 6 (Fig. 2a,b; and Supplementary Fig. 2a). Consistent with previous reports, 99% of the piRNAs are mapped to intergenic regions (64%) or introns (35%) and 1% to exons^34^,^35^ (Supplementary Fig. 2b and Table 1). Of the 555 piRNAs, 260 (47%) are matched in the NCBI (Supplementary Data 1) and 295 (53%) are novel (Supplementary Fig. 3 and Supplementary Data 2). Majority of the novel piRNAs had >100 reads in the samples (Supplementary Fig. 4). Because many of these piRNAs are new, identified in adult tissues and not yet fully characterized, we refer these sncRNAs as piRNA-like sncRNAs or piRNA-Ls in this report.

### Expression patterns of piRNAs and piRNA-Ls

To determine potential biological roles of these sncRNAs, piRNA and piRNA-L expression patterns were first used in an unsupervised hierarchy clustering analysis for the 11 cell lines. NSCLC lines and HBE lines can be clearly clustered together based on the expression patterns of the entire piRNAs and piRNA-Ls (Fig. 2c), as well as piRNAs or piRNA-Ls individually (Fig. 2d,e). Because among the eight NSCLC cell lines, four (H157, H226, H596 and SK-MES-1) derived from patients with lung squamous cell carcinoma (SCC) and four (H522, H1437, H1792 and H 1944) from patients with adenocarcinoma (ADC), we could also examine the patterns between these two major NSCLC subtypes. The expression patterns were in fact able to separate ADC subtype from SCC subtype of NSCLC (Table 1 and Fig. 1d,e). These results suggest that these sncRNAs may play a biological role in lung tumorigenesis and NSCLC differentiation.

**Figure 3:**
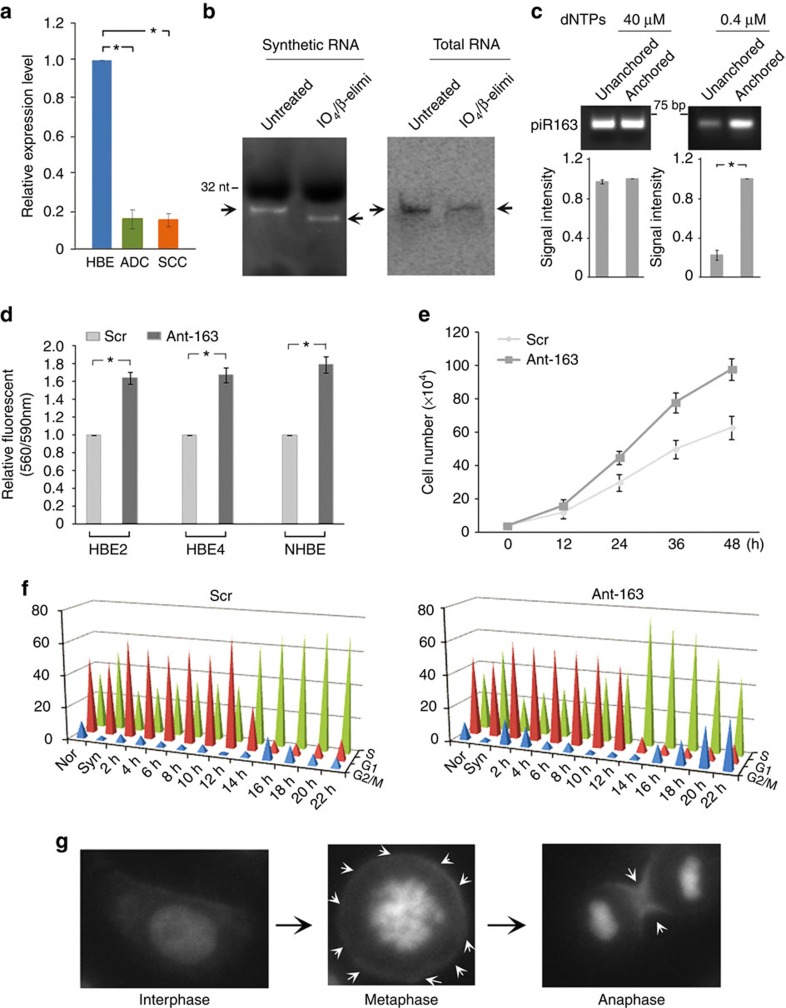
Characteristics of piR-L-163 and its impact in cell cycle. (**a**) Expression levels of piR-L-163 in HBE and NSCLC (ADC and SCC) cells based on RNA sequencing data. (**b**) 2′-*O*-methylation at the 3-termimal of piR-L-163 measured using periodate treatment followed by β-elimination. Synthetic RNAs were separated and stained with ethidium bromide (left). Total RNAs were separated and detected by northern blot using a probe specific for piR-L-163 (right). Arrows indicate piR-L-163 bands. (**c**) The 2′-O-methylation of piR-L-163 was determined by RTL-P. (**d**) Cell survivals after Ant-163 or Scr treatment (48 h). (**e**) Growth of cells treated with Ant-163 or Scr. (**f**) Cell cycle distributions at different time points for HBE4 cells treated with Ant-163 or Scr. (**g**) piR-L-163 distribution at cell cycle phases as merged images of fluorescence *in situ* hybridization and DAPI (4′,6-Diamidino-2-Phenylindole), arrows indicating locations of piR-L-163 . All values are averages of three independent replicates, the error bars reflect mean s.d., and ‘*' indicates *P*<0.01 by Student's *t*-test.

We then analysed differentially expressed piRNAs and piRNA-Ls between NSCLC (ADC or SCC) and HBE cell lines. Using filtered log fold change=1 and false discovery rate <0.05 as criteria, we observed 51 differentially expressed piRNAs or piRNA-Ls between ADC and HBE cells, and 18 between SCC and HBE cells, including nine differentially expressed piRNA-Ls common for both ADC and SCC (Supplementary Table 2). Of these, piRNA-L-163_igs (in brief as piR-L-163) is a piRNA-L aligned to intron 10 of *LAMC2* gene on chromosome 1 and the top commonly downregulated piRNA-L in NSCLC cell lines (Fig. 3a, Supplementary Fig. 5a,b and Supplementary Table 2).

### piR-L-163 and its biological impact in cell cycle regulation

First, we analysed whether piR-L-163 carries a 3-terminal 2′-*O*-methylation, a characteristic feature of piRNA^36^,^37^,^38^,^39^. We used both synthesized oligo RNA and RNA isolated from HBE4 cells and treated these RNAs with periodate (IO_4_) followed by beta elimination and analysed. As expected, the synthesized oligo RNA without 3[prime] 2′-*O*-methylation was sensitive to the IO_4_ treatment and resulted in a two-base reduction (Fig. 3b, left) whereas no reduction was observed for piR-L-163 (Fig. 3b, right), indicating that piR-L-163 carried a 3[prime] 2′-*O*-methylation. We then performed RTL-P (reverse transcription (RT) at low dNTP concentrations followed by PCR)^40^ and showed that the signal intensities of RT–PCR products produced with an unanchored primer was only 20% of the products with an anchored primer when 0.4 μM dNTPs were used (Fig. 3c), further supporting that piR-L-163 had RNA 2′-*O*-methylation consistent with a mature piRNA.

We then wanted to determine whether piR-L-163 plays a functional role in HBE cells. We treated HBE4 cells as well as primary normal HBE (NHBE) cells with piR-L-163 complementary DNA oligonucleotides (Ant-163) or RNA oligonucleotides (Ant-163R) in the experiment. The RNA oligonucleotides triggered a short interfering RNA-like response as expected^41^, and resulted in reducing the expression levels of *LAMC2* but not the DNA oligonucleotides (Supplementary Fig. 5c). We, therefore, used the DNA oligonucleotides in all subsequent experiments as the piR-L-163 blocking agent. Compared with HBE or NHBE cells treated with scrambled DNA oligonucleotides (Scr), the cells treated with Ant-163 showed enhanced cell viability and proliferation (Fig. 3d,e). We next performed cell cycle analysis using HBE4 cells synchronized at S phase followed by treatment with either Ant-163 or Scr. Cell cycle distributions were measured every 2 h. We showed an accelerated DNA synthesis and G2-M accumulation for the cells treated with Ant-163 compared with the cells treated with Scr (Fig. 3f), thereby suggesting a functional role of piR-L-163 in the cell cycle regulation.

We next wanted to know the cellular localization of piR-L-163 in HBE cells. Using a fluorescence *in situ* hybridization method with a digoxin-labelled probe complimentary to piR-L-163, we surprisingly observed that piR-L-163 was not localized in the nucleus as expected for piRNAs^42^, but predominantly located in cytoplasm during interphase, moved into the cell cortex in metaphase and concentrated on junctions of cell division in anaphase (Fig. 3g). The finding is intriguing because piRNAs are believed to function as a PIWI-binding and sequence-dependent epigenetic regulator^1^.

### piR-L-163 binds to p-ERM and regulates p-ERM activity

**Figure 4:**
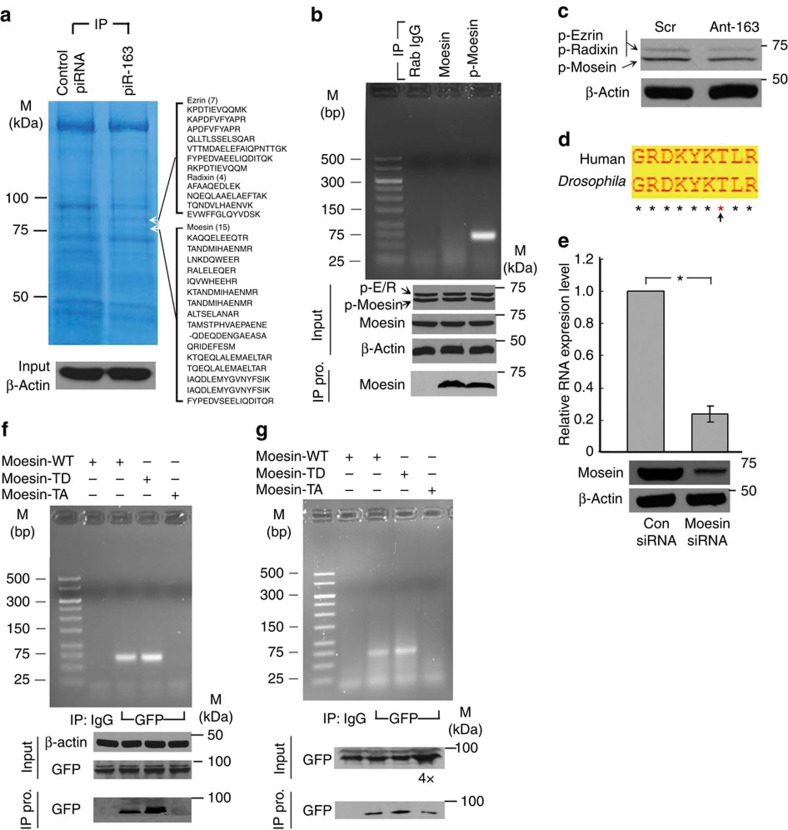
piR-L-163 binds to p-ERM. (**a**) Pulled-down proteins from HBE4 cell lysates with biotinylated scrambled RNA or piR-L-163. Arrows indicate differentially presented bands and detected peptides of ezrin and moesin. (**b**) piR-L-163 detected in RNA purified from various IP products (pro). (**c**) Endogenous p-ERM levels in HBE4 cells. (**d**) Threonine residue in C-terminal of human ERM and *Drosophila* moesin. (**e**) Endogenous moesin was effectively downregulated in HBE4 cells. (**f**,**g**) piR-L-163 detected in RNAs purified from various IP products in conditions as indicated. All values are averages of three independent replicates, the error bars reflect mean s.d., and ‘*' indicates *P*<0.01 by Student's *t*-test.

Because of the unexpected cellular localizations of piR-L-163 during cell cycle progression, we suspected that piR-L-163 plays a biological role through interaction with proteins. To identify potential piR-L-163 interacting proteins, biotin-conjugated piR-L-163 RNA oligonucleotides were used as a bait to pull down cellular binding proteins in HBE4 cells. Compared with proteins pulled down using Scr control RNA, we revealed two protein bands predominantly presented in the piR-L-163 pull-down product (Fig. 4a). Liquid chromatography–tandem mass spectrometry (LC-MS-MS) analysis of the bands revealed ERM proteins as the major protein components, including seven peptides of ezrin, five peptides of radixin and 15 peptides of moesin (Fig. 4a), suggesting that piR-L-163 binds to ERM proteins in HBE4 cells.

To confirm the binding between piR-L-163 and ERM proteins, we performed immunoprecipitation (IP) assays using anti-moesin antibodies followed by RT–PCR using primers specific for piR-L-163. While piR-L-163 was not detectable in the IP products using either a control immunoglobulin G (IgG) antibody or an antibody for non-phosphorylated moesin (non-p-moesin; Fig. 4b), piR-L-163 was readily detected in the IP product using an antibody specific for ERM proteins with phosphorylated threonine (p-Thr) in the C termini of the proteins (p-ERM), which is the functionally active form of ERM proteins^43^,^44^ (Fig. 4b). It should be noted that p-moesin is the predominant p-ERM protein in HBE4 cells as detected by the phosphorylation specific antibody (Fig. 4c), and the protein levels were not significantly changed following treatment with Ant-163 (Fig. 4c). These results suggest that piR-L-163 only binds to functionally activated p-ERM proteins but not the inactive form of ERM.

To validate that piR-L-163 and ERM binding is p-ERM specific, we replaced with an exogenous *Drosophila* wild-type green fluorescent protein (Moe-WT-GFP) moesin that shares an identical C-terminal motif with human moesin containing the p-Thr site^45^, a constitutively active phosphor mimetic mutant (Moe-TD-GFP), or an inactivated mutant (Moe-TA-GFP) at the p-Thr site^43^,^44^ (Fig. 4d) in HBE4 cells. To be effective, the HBE4 cells expressing these transgenes were treated with double-stranded RNA specifically targeting the 3′ untranslated region of the endogenous moesin RNA to reduce the endogenous moesin level (Fig. 4e). Because all the proteins generated from transfected *Drosophila* moesins contained GFP, an anti-GFP antibody was used to pull down the fusion proteins and to determine whether the IP products containing piR-L-163, an indication of their binding with piR-L-163.

piR-L-163 was only detected in the IP products from Moe-WT-GFP- and Moe-TD-GFP-transfected cells, but not from Moe-TA-GFP-transfected cells (Fig. 4f), thereby supporting the notion that the threonine phosphorylation is critical for the binding between piR-L-163 and moesin. However, we noticed that the amount of Moe-TA-GFP pull-down in the IP product was much lower than that of Moe-WT-GFP (Fig. 4f). It is likely due to difference in the protein conformation of Moe-TA-GFP because the mutation prevented the specific amino acid from phosphorylated, which resulted in a weakened binding affinity between the fusion protein and the anti-GFP antibody. It is also possible that the lack of detectable piR-L-163 in the PI product from Moe-TA-GFP-transfected cells was simply owing to the lower amount of Moe-TA-GFP in the IP product. To rule out the later possibility, we used fourfold input of the PI product from Moe-TA-GFP-transfected cells and repeated the experiment. With a comparable amount of pulled-down Moe-TA-GFP compared with Moe-WT-GFP (Fig. 4g), piR-L-163 remained undetectable in the PI product from Moe-TA-GFP-transfected cells (Fig. 4g), confirming that the lack of detectable piR-L-163 was not owing to the amount of Moe-TA-GFP in the IP product.

### Motif and element critical for piR-L-163 and ERM interaction

**Figure 5:**
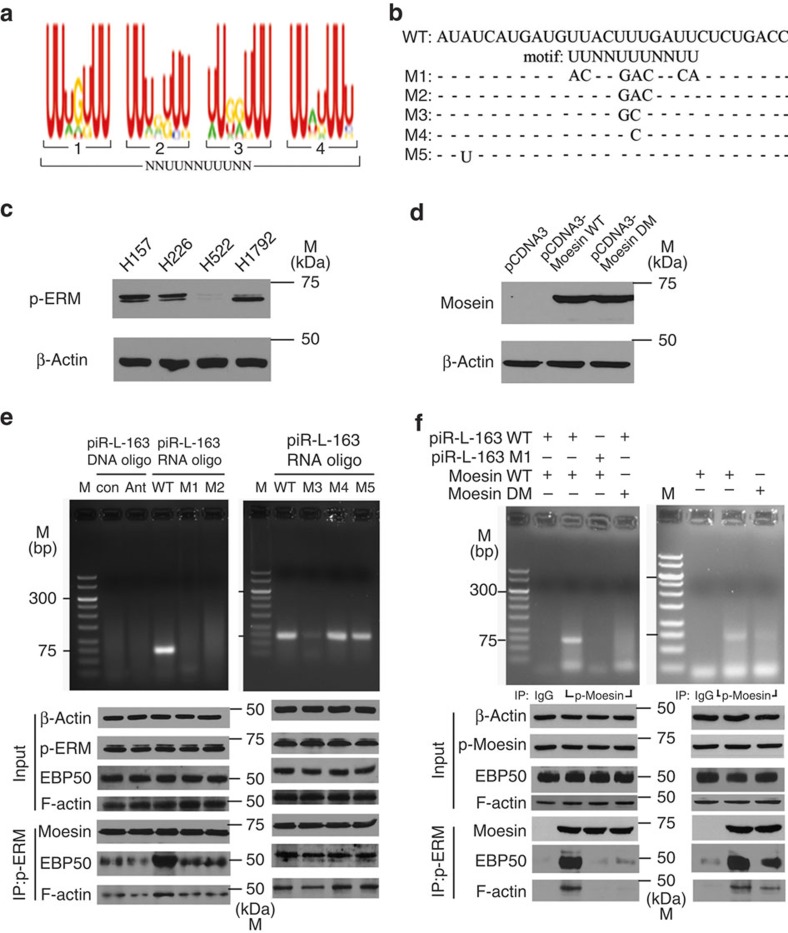
piR-L-163 motif and ERM element critical for binding and p-ERM's interaction with EBP50 and F-actin. (**a**) Predicted protein binding motif NNUUNNUUUNN in piR-L-163. (**b**) Sequences of the mutant piR-L-163s. (**c**) Endogenous p-ERM levels in four NSCLC cell lines. (**d**) Moesin levels in H522 cells transfected with pCDNA3 (empty vector), pCDNA3-moesin WT (WT moesin) and pCDNA3-moesin DM (RRRKPDT element deleted). (**e**) piR-L-163 and its mutant forms detected in RNAs purified from the IP products and the correlated binding capabilities between p-ERM and EBP50 or F-actin in H1792 cells. (**f**) piR-L-163 or piR-L-163M1 detected in RNAs purified from IP products (pro) of H522 cells (left) or HBE4 cells (right) transfected with p-moesin in conditions as indicated and the correlated binding capabilities between p-ERM and EBP50 or F-actin.

We next wanted to determine the site critical for piR-L-163's binding to p-ERM. We first analysed the piR-L-163 sequence and identified a candidate motif ‘UUNNUUUNNUU' with potentially critical for the binding (Fig. 5a). We then generated five mutant forms of piR-L-163 named as M1–M5 (Fig. 5b and Supplementary Data 3) to test their potential impact on the p-ERM binding. For this experiment, we used H1792 cells because these cells expressed extremely low level of endogenous piR-L-163 but considerable amount of p-ERM (Fig. 5c and Supplementary Fig. 5a). H1792 cells were transfected with Scr (control), Ant-163, piR-L-163 (WT) or mutant 1–5 (M1–M5) RNA oligonucleotides, respectively. An anti-p-ERM antibody was used for IP followed by RT–PCR using primers specific for Scr, Ant-163, piR-L-163 or M1–M5 (Supplementary Data 3) to detect the corresponding oligonucleotides. As shown in Fig. 5e, while strong RT–PCR band was detected in the cells transfected with piR-L-163, no RT–PCR band could be detected in the IP products from the cells transfected with Scr, Ant-163, M1 or M2, indicating these oligonucleotides do not bind p-ERM. Conversely, RT–PCR bands with expected sizes were detected in the IP products from cells transfected with M3, M4 or M5, although the band from the M3-transfected cells was substantially weaker than those from M4- and M5-transfected cells (Fig. 5e). These results indicate that the central 3 nucleotides (nt) of the UUNN*UUU*NNUU motif (Fig. 5b) are critical for piR-L-163 to bind p-ERM.

To determine potential element of ERM proteins critical for their binding with piR-L-163, we analysed human and *Drosophila* moesin sequences using BindN ( http://bioinfo.ggc.org/bindn/) and identified RRRKPDT at position 293–299 as a candidate RNA binding element (Supplementary Fig. 6a,b). We then constructed plasmids containing either WT human moesin (moesin-WT) or a mutant form of moesin with RRRKPDT deletion (moesin-DM). Both plasmids produce high levels of moesin proteins in the transfected cells (Fig. 5d). We used two cell lines (H522 and HBE4) to test whether this element contributes to the binding between moesin and piR-L-163. H522 cells expressed extremely low level of endogenous p-ERM (Fig. 5c) and very low level of piR-L-163 (Supplementary Fig. 5a). H522 cells transfected with piR-L-163 were simultaneously transfected with either moesin-WT or moesin-DM to determine the potential binding ability of the introduced moesin proteins with piR-L-163. We also used the non-functional piR-L-163M1 mutant to replace piR-L-163 in the experiment as a negative control. While moesin-WT bound to piR-L-163 but not piR-L-163M1 as expected (Fig. 5f, left panel), moesin-DM failed to bind piR-L-163 (Fig. 5f, left panel), indicating that the RRRKPDT element is critical for moesin's interaction with piR-L-163. We next transfected HBE4 cells that expressed both p-ERM and piR-L-163 (Fig. 4e and Supplementary Fig. 5a) with either moesin-WT or moesin-DM after knocking down the endogenous moesin in the cells. We demonstrated again that only moesin-WT but not moesin-DM bound piR-L-163 (Fig. 5f, right panel).

### piR-L-163 is critical for ERM's functional activities

Because p-ERM functions primarily through binding to F-actin and EBP50 through their C-terminal ERM association domain (C-ERMAD) and four point one ezrin radixin moesin (FERM) domain^19^,^21^,^46^, we analysed the binding affinities of p-ERM with both EBP50 and F-actin in IP products from H1792 cells treated with Scr, Ant-163, WT or M1-M5. As expected, the ability of p-ERM to bind F-actin and EBP50 was closely correlated with binding of piR-L-163 and p-ERM (Fig. 5e). In H522 cells, WT moesin (moesin-WT) together with piR-L-163 but not the mutant piR-L-163 (piR-L-163M1) nor a mutant moesin (moesin-DM) resulted in an enhanced binding with F-actin and EBP50 (Fig. 5f). These results indicate that piR-L-163 binds moesin and is critical for moesin's ability to interact with F-actin and EBP50.

**Figure 6:**
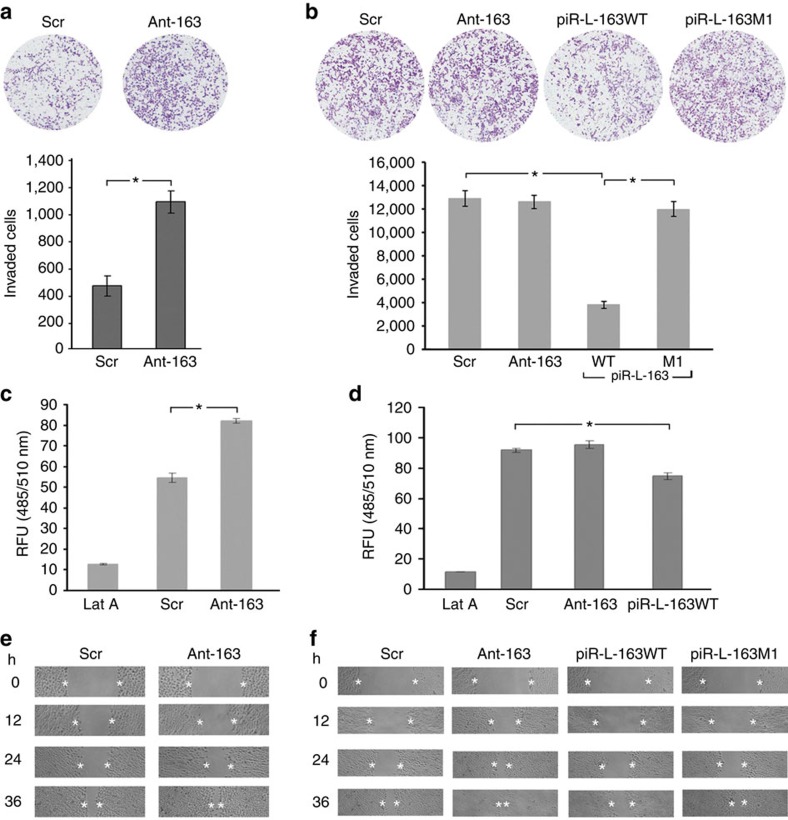
piR-L-163 affects migration and invasion. (**a**) Trans-well assay showed increased invading cells after treating HBE4 cells with Ant-163 compared with cells treated with Ant-163. (**b**) In H1792 cells (extremely low endogenous piR-L-163 expression), piR-L-163 significantly reduced the number of invading cells, but no impact of Ant-163 or piR-L-163M1 treatment was seen. (**c**) Migration capability of HBE4 cells treated with either Ant-163 or Scr measured by a quantitative assay. (**d**) Migration capability of H1792 cells treated with piR-L-163, Ant-163 or Scr. Lat A served as an internal control for background. (**e**) Gap closure of HBE4 cells treated with either Ant-163 or Scr. (**f**) Gap closure of H1792 cells treated with Ant-163, piR-L-163, piR-L-163M1 and Scr. All values are averages of three independent replicates, RFU (relative fluorescence unit), the error bars reflect mean s.d., and ‘*' indicates *P*<0.01 by Student's *t*-test.

We then tested the potential impact of piR-L-163 in cell migration and invasion, two of the properties involving ERM's biological functions^11^,^47^,^48^,^49^. In a trans-well invasion assay, HBE4 cells treated with Ant-163 showed a significantly increased invasion capability compared with cells treated with Scr control, even after 15% downward adjustment for the cells treated with Ant-163 to compensate the possible increase of the cell number after 12 h (Fig. 6a). Conversely, H1792 cells transfected with piR-L-163 exhibited a significantly decreased invasion capability compared with the cells treated with a control RNA oligonucleotides (Fig. 6b). The cells transfected with either Ant-163 or a mutant piR-L-163 showed no impact to the cells' invasion capability (Fig. 6b).

For cell migration, we used two complementary assays. In a quantitative cell migration assay, HBE4 cells treated with Ant-163 exhibited significantly increased cell migration compared with the cells treated with Scr control (Fig. 6c). Conversely, H1792 cells transfected with piR-L-163 showed significantly decreased cell migration compared with the treatment of cells with Scr or Ant-163 (Fig. 6d). To compensate potential differences in the cell numbers among different treatment conditions, we seeded 10% less cells for Ant-163-treated HBE4 cells and 10% more cells for piR-L-163-transfected H1792 cells. In the wound-healing assays, HBE4 cells treated with Ant-163 showed a faster gap closure than the cells treated with Scr control (Fig. 6e). Conversely, H1792 cells transfected with piR-L-163 showed a slower gap closure compared with cells transfected with Scr control, Ant-163 or a mutant piR-L-163 (Fig. 6f).

## Discussion

In this study, we systematically profiled the expression of piRNAs and piRNA-Ls in adult human airway cells including both immortalized NHBE and lung cancer cells. Because piRNAs are predominantly expressed in germline cells to play a key role in suppressing activities of transposons, the identification of >550 piRNAs or piRNA-Ls in these adult cells is important, suggesting that these sncRNAs play biological roles beyond transposon regulation. Because we used a conservative calling (≥20 reads in a cell line) and the relatively low total reads (4.5 million) for the 11 cell lines, the actual number of piRNAs/piRNA-Ls expressed should be higher. Further studies will be needed to determine whether these piRNAs/piRNA-Ls are biologically important in the adult cells, particularly those expressed at very low levels.

Nevertheless, our data suggest that these piRNAs/piRNA-Ls play certain biological roles and involve in lung tumorigenesis because the expression patterns are distinctive between NHBE and lung cancer cells. Furthermore, different expression patterns were also observed between ADCs and SCCs. Although this study did not focus on potential applications of piRNAs/piRNA-Ls for patients with lung cancer, our data suggest that piRNAs/piRNA-Ls may be a new class of molecules potentially useful as biomarkers for cancer classification as well as therapeutic targets.

The finding that piR-L-163 binds directly to p-ERM and regulates ERM functional activities is unexpected and mechanistically important. Our data demonstrate a dynamic interaction between piR-L-163 and p-ERM in subsequent functional activities reflected to cell proliferation, migration and invasion. It should be noted, however, that other factors might also be involved in addition to ERM for piR-L-163-mediated impact in cell proliferation, migration and invasion. Further studies will be necessary to address these issues. Nevertheless, this is the first time that a sncRNA is revealed to participate in a protein functional regulation through a direct interaction with the protein in mammalian cells, in this case, through piR-L-163 binding to p-ERM. We have narrowed down the critical RNA motif in piR-L-163 to the central 3 nt (UUNN*UUU*NNUU) and a small peptide element in ERM critical for the interaction. Based on current model, upon ERM proteins bind to ptdIns(4,5)P2 (PIP2), which is required for phosphorylation of the threonine in the C terminus of ERM^11^,^12^,^16^,^50^, the bound between FREM and C-ERMAD domains weakens leading to the clamp formed by the two domains opens. It is therefore possible that piR-163 binds to p-ERM at this point to stabilize the opening structure, and allow the binding sites in FREM and C-ERMAD domains interacting with the cytoplasmic tail of EBP50 and F-actin.

Our study raises more questions than answers. One important question is the potential biogenesis of piRNAs/piRNA-Ls in these somatic cells. Although expression and potential functions of piRNAs have been suggested in somatic tissues, such as in *Drosophila* ovary and testis (Lin review), their biogenesis and functional mechanisms remain largely unknown. It has been reported that some PIWI proteins were detected in certain somatic tissues including cancer tissues^10^,^51^,^52^,^53^, suggesting that the piRNAs detected in these tissues might be generated in ways similar to germline cells. Another important question is whether the mechanism of piR-L-163 we revealed in this study is an exception for sncRNAs or a common mechanism in mammalian physiology and pathophysiology. If later is true, future studies may lead to a paradigm change in our understanding of biological roles and mechanisms of sncRNAs human physiology and diseases.

## Methods

### Cell culture

Human NSCLC cell lines H157, H226, H596, SK-MES-1, H522, H1437, H1792 and H1944 were obtained from American Type Culture Collection (ATCC) (Manassas, VA, USA) and cultured in RPMI 1640 with 10% fetal bovine serum (FBS) (Sigma-Aldrich). Human HBE cell lines (HBE2, HBE3 and HBE4) were provided by Dr John D. Minna (University of Texas Southwestern Medical Cancer, Dallas, Texas) and cultured in keratinocyte-serum-free medium (SFM) with L-glutamine, prequalified human recombinant epidermal growth factor and bovine pituitary extract (BPE) (Life Technologies). All the cell lines were genotyped for their authentication (the test was done on 24 October 2014). Mycoplasma contaminations were regularly tested and the cells were routinely treated to prevent mycoplasma growth.

### Total mature piRNA purification

To obtain purified mature piRNAs, sncRNAs (<200 nt) were at first separated from total RNA, and then piRNAs were purified from sncRNA in 1 nt resolution gel. The entire process consisted of total RNA extraction, sncRNA separation, piRNA separation and piRNA enrichment. For total RNA extraction, mirVana miRNA isolation kit (Ambion) was used according to the manufacturer's instructions. For sncRNA separation, extracted total RNAs were separated using Craig C. Mello Lab's sncRNA cloning protocols (Gu W and Conte D. http://www.umassmed.edu/uploadedFiles/nemo/Mello%20lab%20small%20RNA%20cloning%20protocol.pdf) with following minor modifications: mixed 80 μl (≤1 mg) of total RNA, 400 μl (5 × volume of total RNA) of mirVana lysis/binding buffer and 48 μl (1/10 volume of total RNA and lysis/binding buffer) of mirVana homogenate buffer in a 1.5-ml Eppendorf (EP) tube; incubated the tube at room temperature for 5 min to denature RNA followed by adding 1/3 volume (176 μl) of 100% ethanol and mixed well; span the tube at 2,200*g* for 4 min at room temperature to remove larger (>200 nt) RNA, and then transferred the supernatant to a new EP tube and added isopropanol (∼700 μl); precipitated sncRNAs at −80 °C until it was frozen (∼30 min) and pelleted sncRNA at 20,000*g* at 4 °C for 40 min; and washed once with 70% cold ethanol (American Bioanalytic) and dissolved the pellet with nuclease-free water.

Next, piRNAs were separated on 15% denaturing acrylamide gel with following specific steps:

Gel was prepared by mixing the following reagents in 50 ml tube: 6.3 g urea (Fisher Scientific), 1.5 ml 10 × Tris/Borate/EDTA (TBE) (Life Technologies), 5.6 ml 40% acrylamide (Bio-Rad) and 3 ml nuclease-free water (Quality Biological); stirred at room temperature until urea is completely dissolved, then 75 μl of 10% ammonium persulfate (AP) (Sigma-Aldrich) and 15 μl of TEMED (Bio-Rad) were added, mixed well and loaded into the electrophoresis shelf to form gel; pre-run gel in 1 × TBE for 20 min at 300 V; heated samples and markers at 75 °C for 5 min, and put them immediately on ice; run the samples at 300 V for 35 min; and stained the gel with 0.5 μg ml^−1^ ethidium bromide (Sigma-Aldrich) in a clean container for 2–3 min, carefully cutting out the target band with a scalpel using ultraviolet light for visualizing bands.

piRNAs were pelleted using a modified protocol of True Small RNA for sequence preparation (Illumina); punctured the bottom of a sterile, nuclease-free, 0.5-ml microcentrifuge tube 3–4 times with a 21-gauge needle; placed the target band into punctured tube, and put the tube into a new 1.5-ml EP tube; centrifuged the stacked tubes in 20,000*g* in a microcentrifuge for 2 min at room temperature to move the gel through the holes into the 1.5-ml tube, and ensured that the gel was completely moved into the bottom tube; added 300 μl of nuclease-free water to the gel debris, and eluted the DNA by shaking the tube overnight at room temperature followed by transferring to the top of a 5-μm filter, and centrifuged the filter for 10 s in 600*g*; added the following reagents to the collected fluid: 2 μl glycogen (Invitrogen), 30 μl 3 M NaAc (Invitrogen) and 975 μl pre-chilled 100% ethanol; precipitated at −80 °C until it was frozen and then pelleted by 20,000*g* at 4 °C for 40 min; and washed once with 70% cold ethanol and dissolved the pellet in nuclease-free water.

### Prepare piRNAs for sequencing

As described in Fig. 1, 5′- and 3′-end adaptors containing barcodes were added to extracted sncRNAs. RT–PCR was performed according to the manufacturer's instructions of True Small RNA kit. RNA sequencing was performed using Illumina sequencer in the University of Maryland Institute for Genome Sciences.

### Periodate treatment and beta elimination

The method was used to determine 2-*O*-methylation at the 3′-end^54^,^55^. Synthetic 30 bases RNA without modification was used as a control. Briefly, sncRNAs from HBE4 cells or synthetic RNA was dissolved in 25 μl of 10 mM NaIO_4_, kept at 4 °C for 40 min in dark room; RNAs were precipitated and the pellet was dissolved in 60 μl of 1 M L-lysine (pH 8.5, Sigma-Aldrich) and kept the tube at 45 °C for 90 min; RNAs were precipitated and dissolved in nuclease-free water; separated the RNAs on 15% denaturing acrylamide gel; for positive control, stained the gel with ethidium bromide in a clean container for 2–3 min, then took photos (UVP, BioSpectrum AC Imaging System); for sncRNAs extracted from HBE4 cells, transferred the gel to Zeta-Probe Membrane (Bio-Rad) in 0.5 × TBE for 60 min at 80 V for northern blot after ultraviolet crosslinking (UV Stratalinker 2,400, Stratagene).

### Northern blot

Probe for detecting piR-L-163 was 5′-GGTCAGAGAATCAAAGTAACATCATGATAT-3′ (synthesized by Integrated Device Technology). Chemically synthesized oligonucleotides were labelled with γ-^32^P-ATP with T4 polynucleotide kinase (Thermo Scientific). Briefly, separated labelled oligonucleotides from unincorporated label by gel filtration on NucAway Spin Columns (Ambion) and 10 k c.p.m. of ^32^P-labelled oligonucleotides were used for each reaction; Pre-wash the membrane in 0.1 × SSC (Saline-Sodium Citrate) (Quality Biological) with 0.1% SDS (Bio-Rad) for 1 h at 65 °C; removed pre-wash solution, preheated pre-hybridization buffer (Ambion) to 40 °C and then hybridized the membrane in the buffer for 2 h at 40 °C; removed pre-hybridization buffer, added labelled probe to 10 ml hybridization buffer (Ambion) and hybridized overnight at 37 °C; washed blot in 6 × SSC with 0.1% SDS for 5 min at room temperature for three times and pre-erased phosphorlmager screen simultaneously for 20 min on light table; repeated wash a fourth time for 20 min at 30 °C, laid the damp blot on clean saran wrap after finishing wash, and fold wraps to seal blot; exposed wrapped blot to phosphorlmager screen in cassette and imaged screen after 2 h.

### Reverse transcription–PCR

To amplify sncRNAs, we added an adaptor to the 3′-end, a process including adaptor ligation, RT and PCR^56^,^57^. For adaptor ligation, the adaptor/5′rapp/5′-CTGTAGGCACCATCAAT-3′/3′ddc/with both 5′ and 3′ modification was used. Briefly, mixed 1 μl (10 pmol, ∼55 ng) of RNA 3′ adaptor and 4.2 μl (∼100 ng) of purified sncRNAs in a 1.5-ml EP tube; incubated at 72 °C for 2 min, then placed on ice immediately and kept it on ice for at least 1 min; added the following reagents (New England Biolabs) to the above tube: 0.8 μl 10 × RNA ligase buffer, 1 μl RNase inhibitor and 1 μl single-strand RNA ligase; mixed it well and incubated at 37 °C for 1 h, then terminated the reaction at 65 °C for 15 min. For RT, SuperScript III First-Strand Synthesis System (Invitrogen) was used according to the manufacturer's instructions with gene-specific primer (5′-CAAGCAGAAGACGGCATACGAATTGATGGTGCCTACAG-3′). For PCR, a common reverse primer (5′- CAAGCAGAAGACGGCATACGA-3′) and primers specific for individual sncRNAs (Supplementary Data 3) were used. Amplification conditions were denaturation at 95 °C for 30 s (5 min for the first cycle), annealing at 60 °C for 20 s and extension at 72 °C for 20 s (2 min for the last cycle) for 25 cycles.

### Immunoprecipitation

For IP using piR-L-163, synthesized biotin-labelled RNA oligonucleotides (/5′Biosg/5′-AUAUCAUGAUGUUACUUUGAUUCUCUGACC-3′) was used and scrambled RNA (/5′Biosg/5′-GAUACCAAGGACAUACGCUUAUGCAUGCUA-3′) was used as a control.

Protein extracts from 1 × 10^7^ cells using HKMG lysis buffer (10 mM HEPES, pH 7.9, 100 mM KCl, 5 mM MgCl_2_, 10% glycerol, 1 mM dithiothreitol and 0.1% NP40) with protease and phosphatase inhibitors (Roche) were incubated with 1 μg biotin-labelled RNA for 16 h at 4 °C with rotation after pre-clearing with streptavidin beads, then coupled to 10 μl 50% of streptavidin agarose beads (Sigma-Aldrich). After incubation, the biotin-labelled oligonucleotide-coupled streptavidin beads were washed four times with HKMG lysis buffer. Samples were denatured in SDS protein loading buffer before running on a SDS–acrylamide gel.

For IP using anti-human moesin or p-ERM antibodies, anti-moesin (clone Q480, catalogue no. 3150, Cell Signaling) or anti-p-ERM (clone 41A3, catalogue no. 3149, Cell Signaling) antibodies were used, and a rabbit IgG was used as a control. Briefly, protein G agarose beads (Sigma-Aldrich) were saturated with rabbit IgG, anti-moesin or anti-p-ERM antibodies, respectively (1 μg antibody was used for 4 μl agarose beads) at 4 °C for 3 h, centrifuged at 2,200*g* for 5 min and discarded the upper aqueous phase; protein extracts prepared from cells using lysis buffer (20 mM Tris-cl, pH 7.6, 150 mM NaCl, 20 mM KCl, 1.5 mM MgCl_2_, 0.5% NP-40, 0.5 mM PMSF) were pre-cleared using protein G agarose at 4 °C for 2 h, centrifuged at 2,200*g* for 5 min, and transferred the upper aqueous phase to above tubes with agarose beads saturated with antibodies, respectively; Kept the tubes rotating overnight at 4 °C, centrifuged at 2,200*g* for 5 min, and washed the beads with lysis buffer twice; Extracted total RNAs from beads using Trizol Reagent (Life Technologies); Amplification of piR-L-163 or its mutants was performed according to the steps described in the ‘Reverse transcription–PCR' section.

For IP using GFP antibody (clone GSN149, catalogue no. G1546, Sigma-Aldrich), moesin mutants of *Drosophila* were used to test piR-L-163 binding ability. Protein extracts were prepared from cells at 48 h after plasmids transfection. GFP-saturated protein-G agarose beads were added to the pre-cleared extracts; RNA precipitation, adaptor ligation, RT and PCR were performed as described above.

### Mass spectrometry

The target band for MS was performed. The specific protocol is as follows: selected gel bands were excised from the one-dimensional SDS–polyacrylamide gel electrophoresis gel, cut into 1 × 1 mm cubes and destained in 50% acetonitrile in 100 mM NH_4_HCO_3_. Gel pieces were reduced with 10 mM Tris (2-carboxyethyl) phosphine hydrochloride Q22 (Thermo Scientific) in 100 mM NH_4_HCO_3_ for 60 min at 56 °C, and were alkylated with 55 mM iodoacetamide (Sigma) in 100 mM NH_4_HCO_3_ for 1 h at room temperature in the dark. After washing, the gel pieces were dehydrated with 100% acetonitrile and dried using a speed vacuum of Q23 1.5 mg of trypsin (Promega), and a volume of 50 mM NH_4_HCO_3_ was added to each gel piece and the gel pieces were allowed to swell for 30 min on ice. Excess trypsin was removed and replaced with 50 mM NH_4_HCO_3_, and samples were incubated at 37°C overnight. The resulting peptides were extracted using 2.5% formic acid and 50% acetonitrile in 50 mM NH_4_HCO_3_.

The processed samples were analysed using a nanoscale reversed-phase LC using an Xtreme Simple nanoLC system (CVC/Q24 MicroTech). The analytical column was prepared by packing into a laser-pulled-fused silica capillary, and peptides were injected into the sample loop using an Endurance auto sampler. A 120-min LC-gradient method with a post-split flow rate of 0.6 ml min^−1^ was used to elute Q25 peptides into the LTQ mass spectrometer with a nanospray ionization source. Dynamic exclusion was enabled with repeat count 2, repeat duration 30 s and exclusion duration 120 s. MS and tandem Q26 MS data were searched against the UniProtKB human protein database using Bioworks 3.3.1 SP1 with the SEQUEST algorithm. Search parameters included 1.5 Da peptide mass tolerance, 1.0 Da fragment tolerance, static Cys+57.02510 (carbamidomethylation) modification and differential modification Met+15.99492. Fully tryptic peptides with up to two missed cleavages and charge-state-dependent cross-correlation scores were ≥2.5, 3.0 and 3.5 for 2+, 3+ and 4+ peptides, respectively.

### RNA interference

The small interference RNA was used to target the untranslated region of human moesin (5′-CCGUUAGCAGGAAGCCUAA-3′) with scrambled sequence as a control (5-GAUACCAAGGGACAUACGCUU-3′). Sense and antisense oligo RNAs of moesin and scrambled control were annealed, respectively. Transfection is performed using Lipofectamine^TM^ RNAiMAX (Invitrogen) according to its manual, and the final concentration of annealed oligo RNAs is 400 nM. Transfection and knockdown efficiency were tested at both RNA and protein levels.

### Blocking piR-L-163 expression

To avoid triggering uncertain short interfering RNA^20^, complementary DNA was used as antagonism targeting piR-163 (5′-GGTCAGAGAATCAAAGTAACATCATGATAT-3′) with scrambled DNA (5′-GATACCAGGGACATACGCTTGATCCTAGC-3′) as a control.

### Western blot and antibodies

. Primary antibodies used were: anti-moesin (1:1,000, Clone Q480, catalogue no. 3150, Cell Signaling), anti-p-ERM (clone 41A3, catalogue no. 3149, Cell Signaling), anti-moesin (clone 38/moesin, catalogue no. 610401, BD Transduction Laboratories), anti-p-moesin (Thr558, catalogue no. 12895, Santa Cruz), anti-β-actin (1:5,000, catalogue no. A228, Sigma-Aldrich), anti-EBP50 (1:100, clone 6/EBP50, catalogue no.61160, BD Transduction Laboratories), anti-F-actin (1:100, clone NH_3_, catalogue no. MA1-80729, Thermo Scientific), anti-p-Wee 1 (Se53, 1:1,000, catalogue no. sc-130223, Santa Cruz), anti-Wee 1 (C-20, 1:1,000, catalogue no. sc-325, Santa Cruz), anti-p-Cdc2 (Tyr15, 1:1,000, catalogue no. 9111, Cell Signaling), anti-p-Cdk (Thr14/Tyr15-R, 1:1,000, catalogue no. sc-28435-R, Santa Cruz), anti-Cdc2 p34 (H-297, 1:1,000, catalogue no. sc-747, Santa Cruz), anti-p-Cdc25C (Ser198, 1:1,000, catalogue no. 9529, Cell Signaling), anti-CDK2 (78B2, 1:1,000, catalogue no. 2546, Cell Signaling), anti-p-histone H2AX (Ser139, 20E3, 1,000, catalogue no. 9718, Cell Signaling) and anti-GFP (N-terminal, 1:4,000, catalogue no. G1544, Sigma). Secondary antibodies used were as follows: goat anti-mouse (1:2,500, catalogue no. 31160, Pierce) and goat anti-rabbit (1:2,500, catalogue no. 31460, Pierce). Cells were lysed in RIPA buffer (Sigma) on ice after washing in phosphate-buffered saline (PBS) twice and centrifuging at 160,000*g* for 10 min at 4 °C. Protein in supernatant was qualified using bicinchoninic acid (BCA) protein assay kit (Thermo Scientific) and denatured in SDS loading buffer in boiling water, run 10% SDS–polyacrylamide gel electrophoresis gel and transferred to nitrocellulose membrane (Thermo Scientific). Membrane was incubated with primary antibody overnight at 4 °C after blocking, washed in Phosphate Buffered Saline with Tween® 20 (PBST) and incubated corresponding secondary antibody for 1 h at room temperature, finally it was developed using SuperSignal west pico chemiluminescent substrate (Thermo Scientific). Uncropped scans of the critical western blots presented in Fig. 5 are shown in Supplementary Fig. 7.

### Cell cycle analysis

Cell cycle distribution was analysed by flow cytometry (Becton Dickinson). Cells were synchronized in growth factor-free keratinocyte-SFM with 2 mM thymidine (catalogue no.T1895, Sigma-Aldrich) for 24 h. Transfection is performed using Lipofectamine TM RNAiMAX (Invitrogen) according to its manual, and the final concentration of annealed oligo RNAs is 400 nM. Four hours after transfection, culture medium was replaced by complete keratinocyte-SFM supplemented with L-glutamine, prequalified human recombinant epidermal growth factor (EGF) 1-53 and BPE. Cells were harvested at different time points, washed in PBS, fixed with ice-cold 70% ethanol and strained in PI/RNase solution (BD Pharmingen). The samples were analysed on a FACScan flow cytometer in combination with BD lysis software (Becton Dickinson).

### Cell viability and cell growth curve assay

Cell viability and cell growth curve assay were determined by 3-(4,5-dimethylthiazol-2-yl)-2,5-diphenyltetrazolium bromide (MTT) assay and Trypan blue exclusion assay, respectively. Briefly, specifically, MTT (5 mg ml^−1^) was added into cells and incubated at culture condition for 3 h, removed the medium carefully and dissolved formazan in DMSO, assay was used to assess the surviving cells and optical density (OD) values were measured using Microplate Reader Manager (Bio-Rad) at wavelengths of 570/690 nm. For Trypan blue exclusion assay, cells were washed in PBS for three times and were dyed in 0.4% Trypan blue solution. Unstained viable cells were counted in haemocytometer chamber under microscope. Each experiment was performed three times independently.

### Invasion assay

Briefly, 24 h after transfection, 6 × 10^4^ cells were placed in the upper chamber of Matrigel Invasion Chambers (BD Biosciences) with growth factor-free keratinocyte-SFM, and the bottom chamber was exposed to keratinocyte-SFM medium supplemented with L-glutamine, prequalified human recombinant EGF 1-53 and BPE. Invading cells were evaluated after 12 h according to the manufacturer's instruction. The number of invading cells were adjusted 15% downwards for Ant-163-treated cells or 15% upwards for piR-L-163-treated cells to compensate the differences for the proliferation rates.

### Migration assays

Two complimentary methods were used. For quantitative fluorescent dye assay, Innocyto Cell migration Assay kit (EMD Millipore) was used. Briefly, 24 h after treatment, 1 × 10^5^ cells (10% less for Ant-163-treated cells or 10% more for piR-L-163-treated cells) were loaded to the upper chamber with growth factor-free keratinocyte-SFM for (HBE4 cells) or serum-free DMEM (for H1792 cells) with the bottom chamber exposed to completed culture medium. Negative controls were used with 3 μM latrunculin A. After 12 h incubation, fluorescence was measured using a fluorescence plate reader 485 nm (excitation) and 520 nm (emission) according to the manufacturer's instruction. For wound-healing assay, cells (24 h after treatment with different DNA or RNA oligonucleotides) were grown to confluence followed by scratching with a pipette tip to create a gap. The floating cells were removed, and the gaps were monitored/photographed every 12 h.

### Statistical analysis

The results reported as mean±s.d. indicated in the data sets were analysed using Student's *t*-test under the assumption of equal variance for comparisons. All tests were determined by unpaired two-sided tests, and *P* values <0.05 were considered statistically significant.

### Bioinformatics analysis

The sequencing reads from each of the 11 samples were individually processed and aligned to the human reference (build hg19) using Bowtie 0.12.9 (ref. 58). In-house Perl scripts were used to detect piRNAs expression based on genomic coverage after using a cutoff of 30 × across a minimum length of 26 bp regions. A piRNA prediction tool^59^ was used to filter the detected piRNAs loci from individual samples utilizing the piRNAs sequence. The piRNAs loci from all 11 samples were collated using custom Perl scripts. This merged set of piRNAs loci was further filtered to exclude piRNAs loci that overlapped known protein coding genes and other sncRNAs. Only those piRNAs loci with lengths between 26 and 32 nt were retained as the final set of novel piRNAs loci.

Simultaneously, 32,046 piRNAs nucleotide sequences were queried and downloaded from NCBI. The Blat programme^60^ was used to align these nucleotide sequences against human reference (build hg19) to infer the genomic coordinates for the known piRNA sequences. Blat hits showing 100% alignment against the human reference were retained as the coordinates of the known piRNAs sequences. Known piRNAs sequences that aligned to multiple locations were given separate unique IDs. The read coverage across these known piRNAs loci was calculated using custom Perl scripts. The final set of known piRNAs loci was determined by filtering out piRNAs loci with reads per kilobase per million mapped reads ≤15. Known piRNAs loci that showed partial read support across the entire length were also filtered out.

HTSeq^61^ was used to compute read counts across each piRNA in each of the 11 samples, which in turn were used as input to the R package DESeq^62^. DESeq was used to normalize the read counts for library size and dispersion followed by tests for differential piRNA expression between the immortalized and ADC cell line and the immortalized and squamous cell line. The significant differentially expressed piRNA (the unknown piRNAs are referred as piRNA-Ls in the text) were determined using an false discovery rate cutoff ≤0.05 and at least twofold change between conditions.

### Constructing WT and mutant moesin plasmids

We used pcDNA3.1(+) vector for cloning moesin WT and mutant plasmids. All plasmids were sequenced for verification.

### Interaction between piR163 and moesin

To determine specific piR-L-163 site critical for its binding to moesin, a series of piR-L-163 mutants in the NNUUNNUUUNNUU motif predicted with protein binding capability^63^ were generated. To determine moesin domain critical for its interaction with piR-L-163, a construct was generated with deletion of a candidate RNA binding element (RRRKPDT) based on structures of human and *Drosophila* moesin predicted using software BindN ( http://bioinfo.ggc.org/bindn/).

## Additional information

**Accession codes:** The proteomic data has been deposited in ProteomeXchange Consortium via the PRIDE partner repository with the data set identifier ‘PXD002076'. The piRNA-sequence data are deposited in the GEO database at the National Center for Biotechnology Information under the accession code GSE57681.

**How to cite this article:** Mei, Y *et al.* A piRNA-like small RNA interacts with and modulates p-ERM proteins in human somatic cells. *Nat. Commun.* 6:7316 doi: 10.1038/ncomms8316 (2015).

## Supplementary Material

Supplementary Information Supplementary Figures 1-7 and Supplementary Tables 1-2

Supplementary Data 1 piRNAs matched in NCBI database

Supplementary Data 2 Novel piRNA like small RNAs

Supplementary Data 3 Synthesized DNA and RNA oligonucleotides

## Figures and Tables

**Table 1:** Index sequences for individual cell lines.

**Cell line name**	**Index sequence (5′-3′)**
H157	ATCACG
H226	CGATGT
H596	TTAGGC
SKMES1	TGACCA
H522	ACAGTG
H1437	GCCAAT
H1792	CAGATC
H1944	ACTTGA
HBE2	TAGCTT
HBE3	GGCTAC
HBE4	CTTGTA

Specific sequence tag was added to RNAs of individual cell lines to allow identify the origins of the RNAs for RNA-sequencing analysis.
