# Supplementary material for: A piRNA-like small RNA interacts with and modulates p-ERM proteins in human somatic cells
Source: Nat Commun. 2015 Jun 22;6:7316. doi: 10.1038/ncomms8316 (PMC4557300; doi:10.1038/ncomms8316)
Supplement: Supplementary Information — Supplementary Figures 1-7 and Supplementary Tables 1-2 [file ncomms8316-s1.pdf]

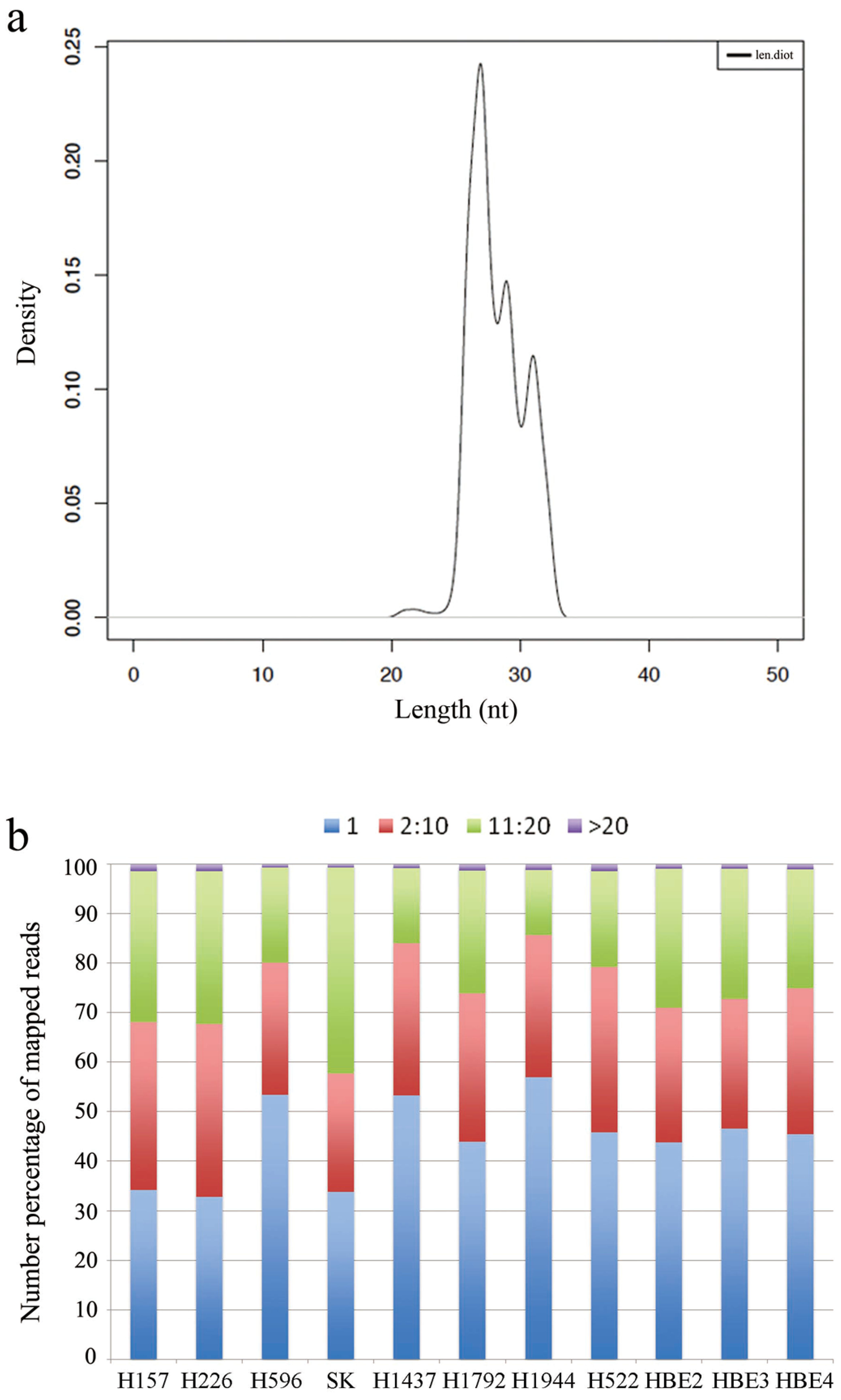


Supplementary Figure 1. Lengths of reads obtained in RNA-seq and human genome distributions of the reads.
(a) Length distribution of all the reads. (b) Numbers of genomic loci mapped for the reads observed in each cell line (percentages of the reads).

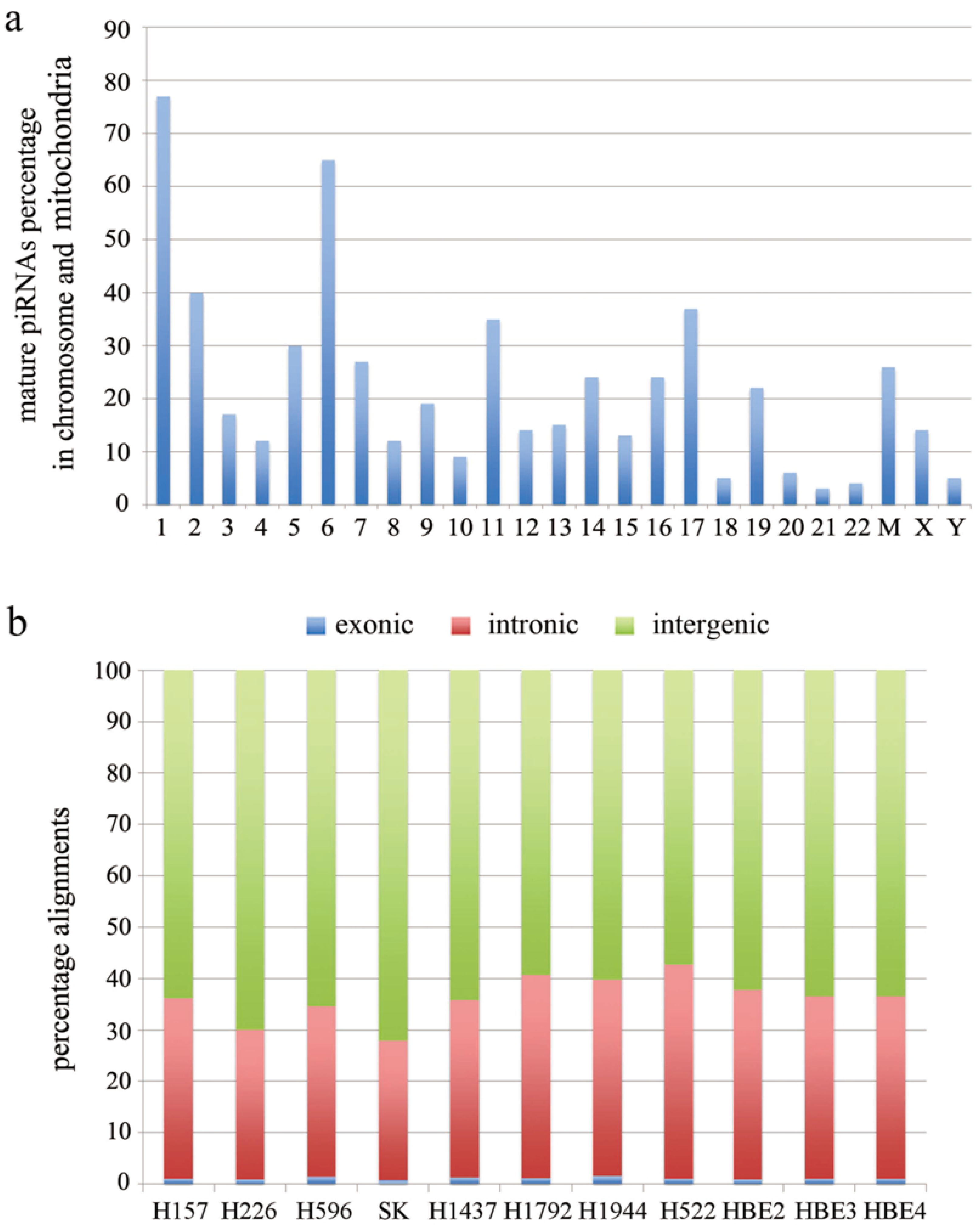


Supplementary Figure 2. Chromosomes and mitochondria genome distributions of the reads. (a) Distribution of all the reads. (b)Percentages of the reads mapped to exonic, intronic, and intergentic regions, respectively for each cell line.

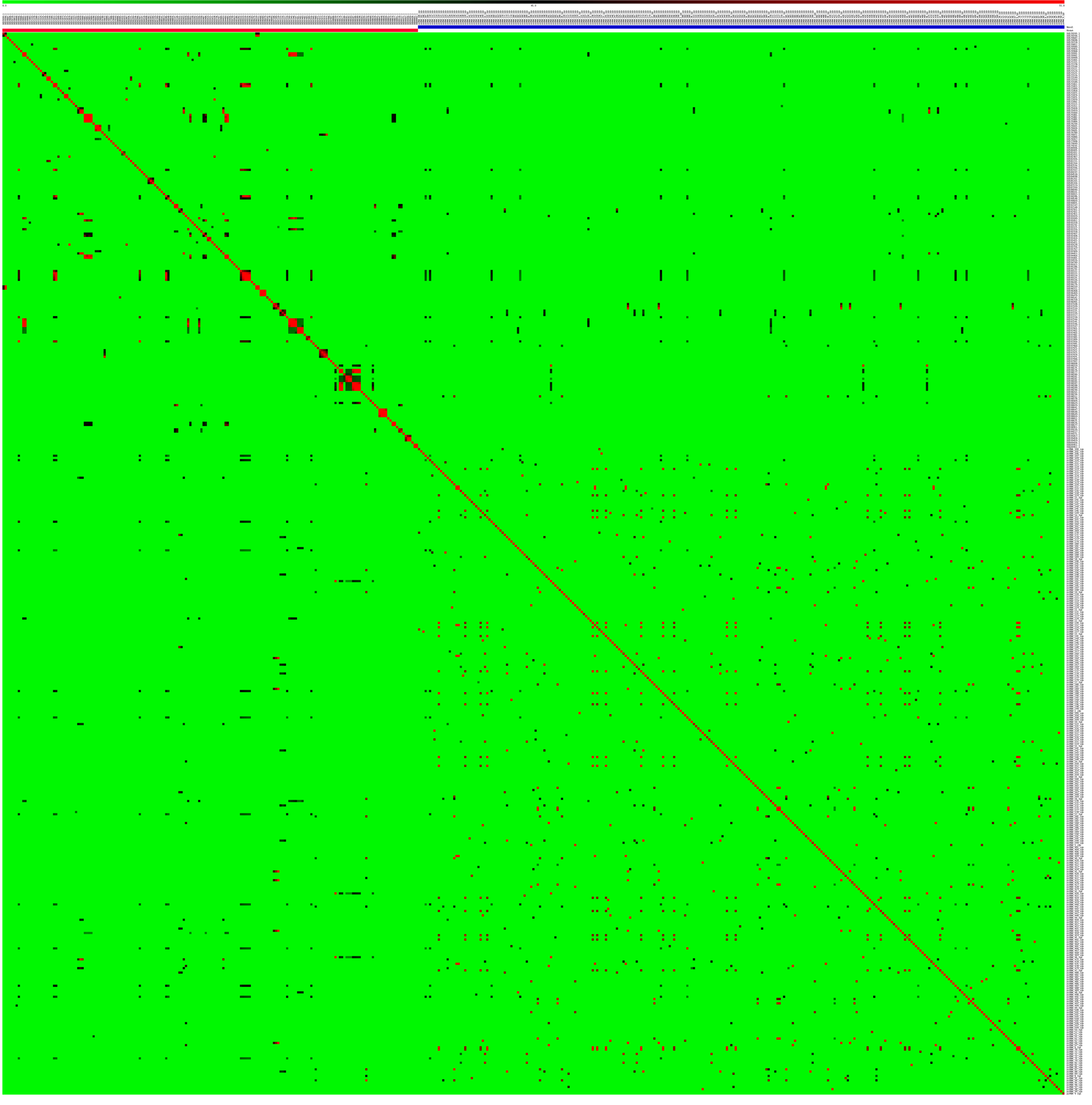

SupplementaryFigure 3. Heatmapof expression of piRNAs and piRNA-Ls identified in the 11 cell lines.

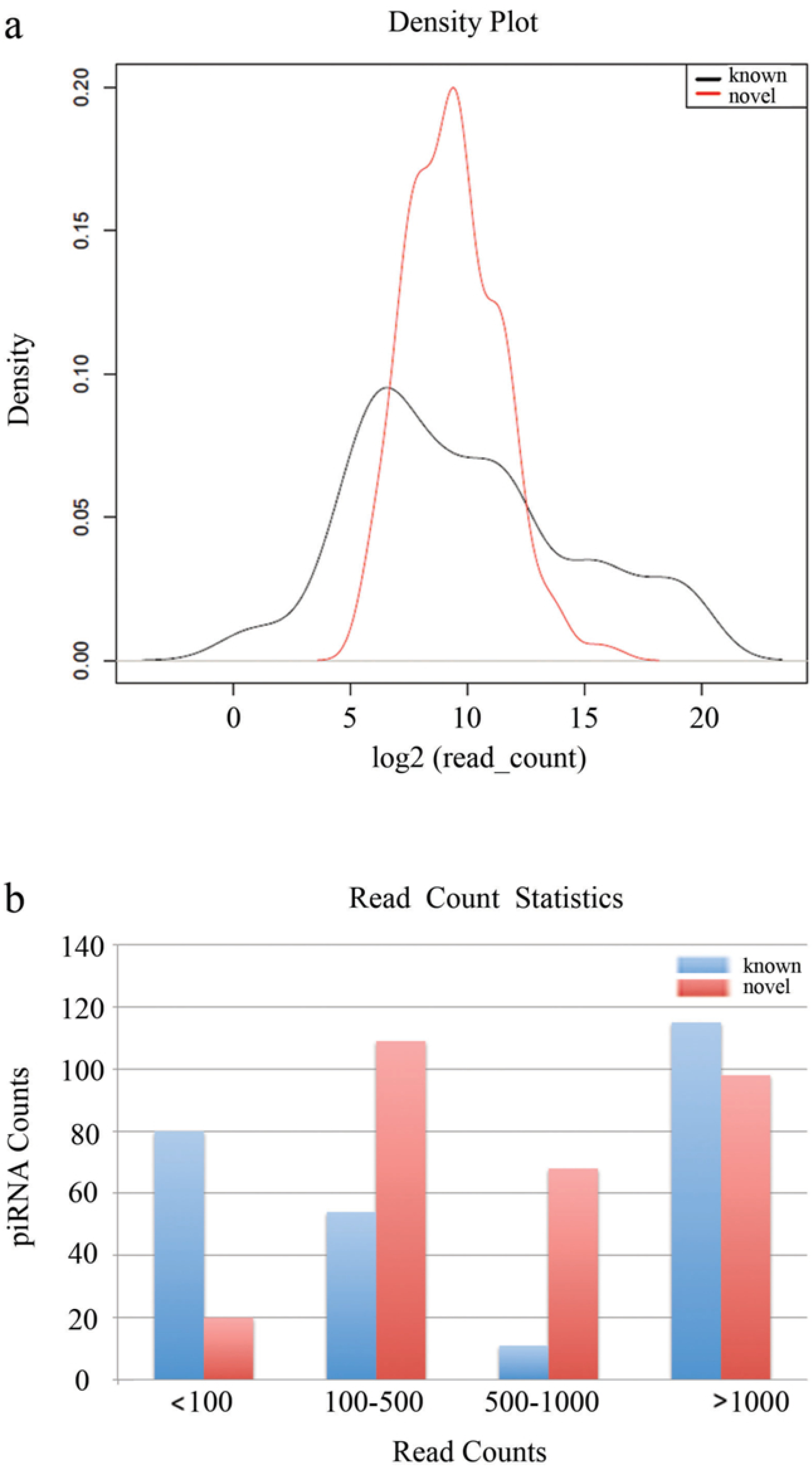


Supplementary Figure 4. Expression levels of piRNAs (known) and piRNA-Ls (novel).
(a) Density plot of all the readsseparated by known and novel. (b) Histogram of average read counts covering the known and novel piRNAs (piRNA/piRNA-L).

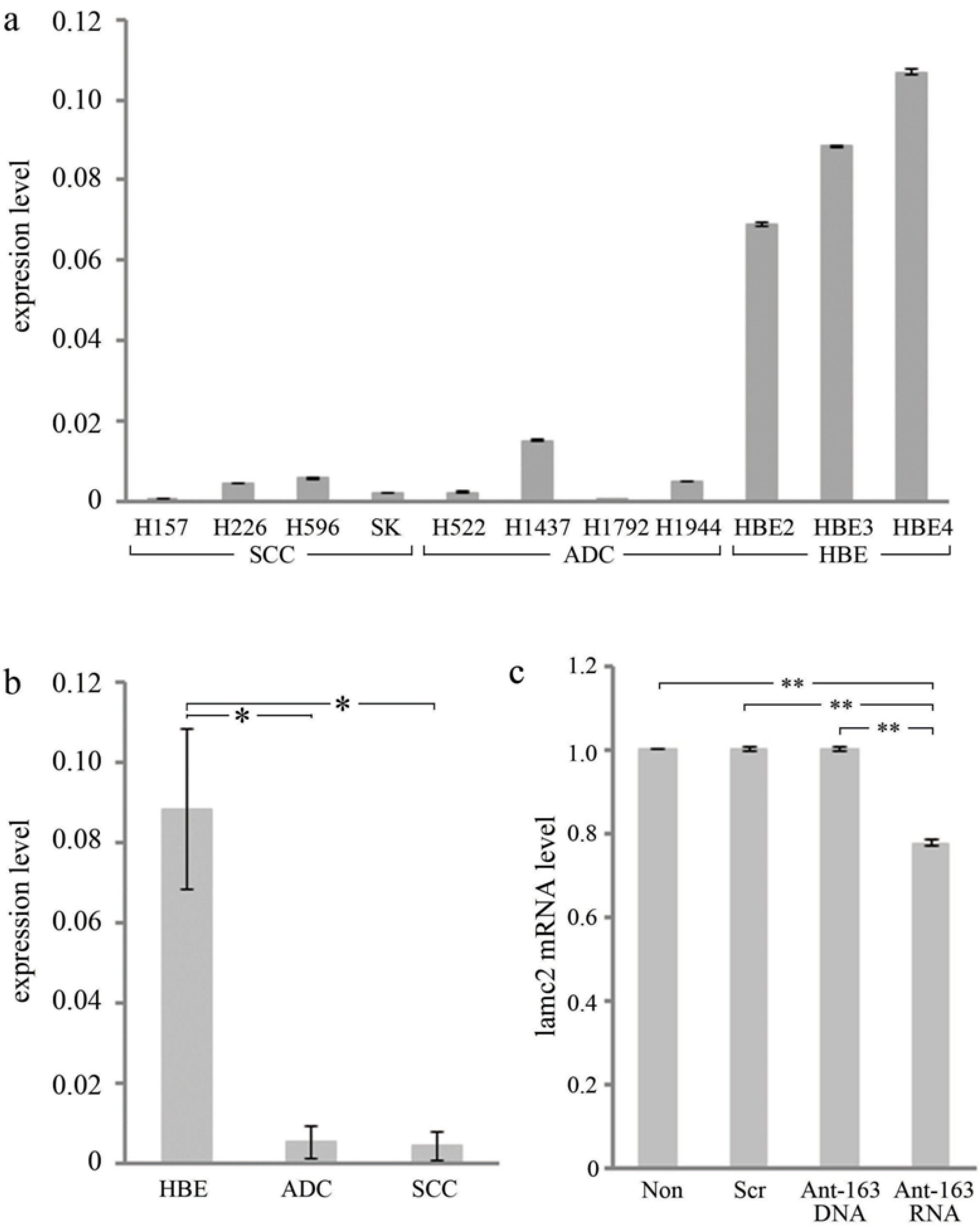


Supplementary Figure 5. Expression of piR-L-163 in NSCLC and HBE cell lines measured by real time RT-PCR.
(a) piR-L-163 expression levels in individual cell lines. (b) piR-L-163 expression levels in HBE, ADC and SCC as groups. All the values are averages of four independent replicates,error bars represent mean s.d., and * indicates $p<0.01$ by Student's t-test.(c) *LAMC2* expression levels in HBE4 cells measured by real time RT-PCR in the conditions as labeled. Values are averages of three independent replicates, error bars represent mean s.d., and ** indicates $p<0.05$ by Student's t-test.

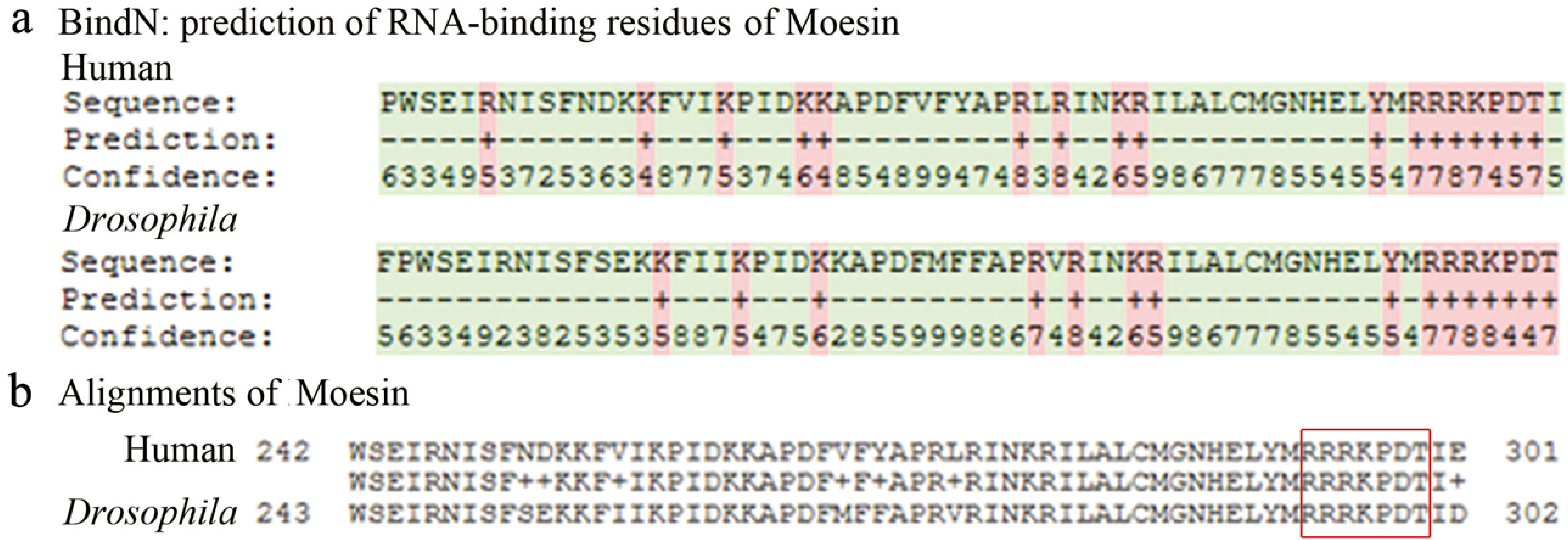


SupplementaryFigure 6. Regions in moesin potentially critical for piR-L-163 and p-ERM interaction. (a) Predicted RNA binding element in human and drosophila moesin. (b) Alignment of human and drosophila moesin.

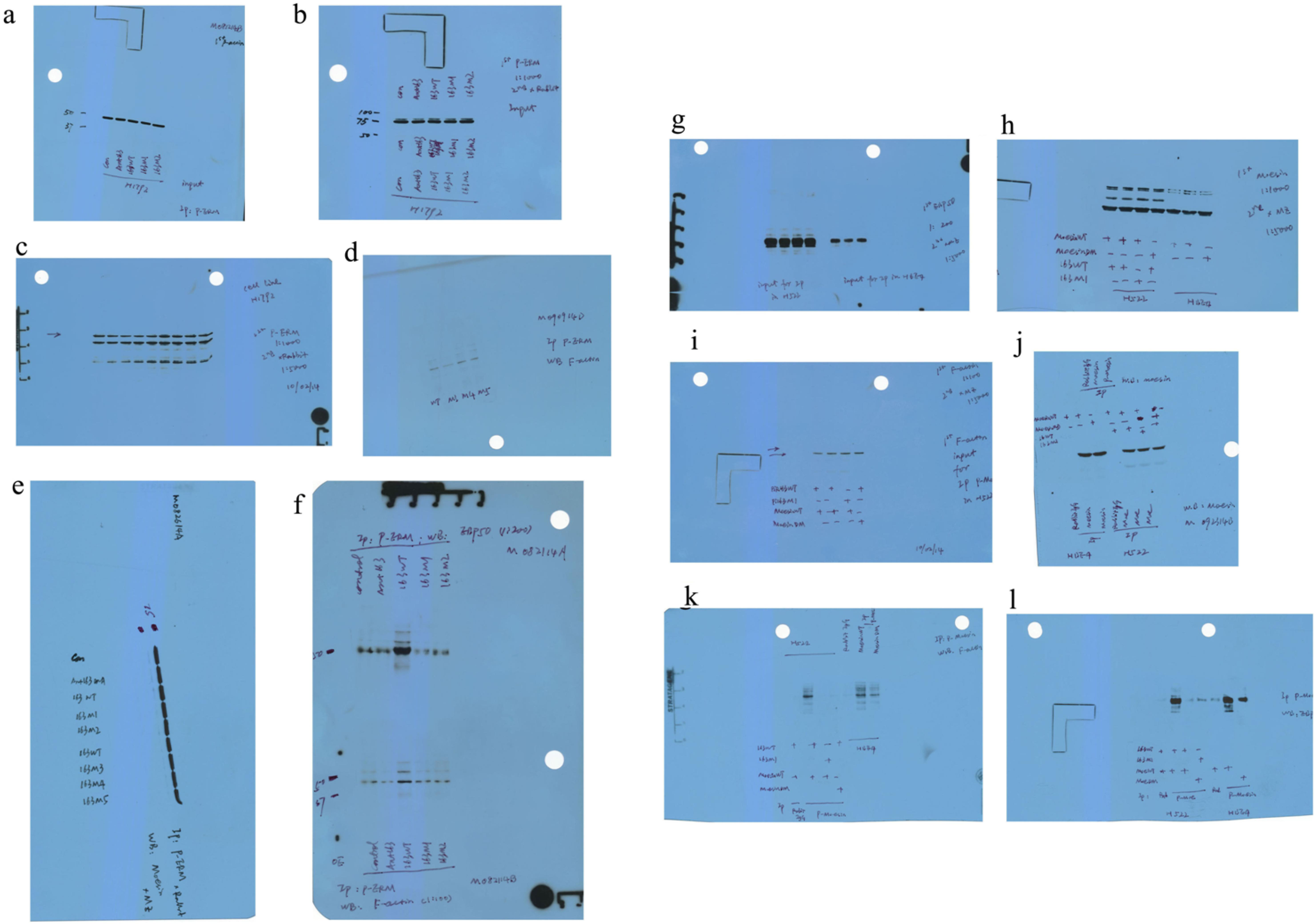


Supplementary Figure 7. Uncropped scans of critical Western blots presented in Figure 5.
(a-c) Protein levels in lysates obtained fromH1792 cells transfected with different oligos. (d-f) p-ERM, F-actin and EBP50 levels in proteins pulled down by p-ERM. (g-h) EBP50 and F-actin levels in lysates of HBE4 and H522 cells with various treatment conditions. (i-l) F-actin, EBP50 and Moesinlevels in proteins pulled down by p-ERM.

Supplementary Table 1. Reads obtained from RNA-seq and their alignments in genome.

| Sample ID | Total Reads | Total Alignments | Exonic (%) | Intronic (%) | Intergenic (%) |
|---|---|---|---|---|---|
| H157 | 412,179 | 6,222,148 | 1 | 35.17 | 64.02 |
| H226 | 429,903 | 4,511,107 | 0.87 | 29.11 | 70.15 |
| H596 | 277,752 | 2,233,230 | 1.34 | 33.19 | 65.81 |
| SKMES1 | 546,528 | 6,310,587 | 0.71 | 27.17 | 72.21 |
| H1437 | 243,863 | 2,310,927 | 1.22 | 34.59 | 64.4 |
| H1792 | 394,242 | 8,701,353 | 1.09 | 39.57 | 59.43 |
| H1944 | 216,476 | 2,254,141 | 1.47 | 38.28 | 60.39 |
| H522 | 711,719 | 18,561,213 | 0.96 | 41.8 | 57.34 |
| HBE2 | 450,773 | 7,491,329 | 0.89 | 36.83 | 62.46 |
| HBE3 | 298,750 | 4,153,110 | 1.05 | 35.44 | 63.68 |
| HBE4 | 508,929 | 6,631,485 | 1.05 | 35.56 | 63.56 |
| | total | | average | | |
| | 4,491,114 | 69,380,630 | 1 | 35 | 64 |

Supplementary Table 2. Differentially expressed piRNAs or piRNA-Ls between ADC or SCC and HBE cells.

| Between ADC and HBE | Between SCC and HBE |
|---|---|
| Feature ID | Feature ID |
| piRNA-L-163_igs | piRNA-L-163_igs |
| piRNA-L-132_igs | piRNA-L-132_igs |
| piRNA-L-133_igs | piRNA-L-133_igs |
| piRNA-L-184_igs | piRNA-L-184_igs |
| piRNA-L-208_igs | piRNA-L-208_igs |
| piRNA-L-216_igs | piRNA-L-216_igs |
| piRNA-L-261_igs | piRNA-L-261_igs |
| piRNA-L-232_igs | piRNA-L-232_igs |
| piRNA-L-409_igs | piRNA-L-409_igs |
| piRNA-37 | piRNA-L-495_igs |
| piRNA-148 | piRNA-L-488_igs |
| piRNA-255 | piRNA-L-138_igs |
| piRNA-L-101_igs | piRNA-L-276_igs |
| piRNA-L-490_igs | piRNA-L-334_igs |
| piRNA-L-124_igs | piRNA-L-408_igs |
| piRNA-L-50_igs | piRNA-127 |
| piRNA-L-505_igs | piRNA-32 |
| piRNA-L-369_igs | piRNA-L-450_igs |
| piRNA-L-381_igs | |
| piRNA-L-130_igs | |
| piRNA-L-87_igs | |
| piRNA-L-40_igs | |
| piRNA-L-181_igs | |
| piRNA-L-212_igs | |
| piRNA-L-89_igs | |
| piRNA-L-112_igs | |
| piRNA-246 | |
| piRNA-L-290_igs | |
| piRNA-L-143_igs | |
| piRNA-L-420_igs | |
| piRNA-L-196_igs | |
| piRNA-142 | |
| piRNA-L-2_igs | |
| piRNA-L-13_igs | |
| piRNA-L-85_igs | |
| piRNA-146 | |
| piRNA-245 | |
| piRNA-L-350_igs | |

piRNA-251
piRNA-L-9_igs
piRNA-238
piRNA-L-97_igs
piRNA-L-404_igs
piRNA-L-257_igs
piRNA-230
piRNA-L-432_igs
piRNA-L-18_igs
piRNA-38
piRNA-243
piRNA-L-188_igs
piRNA-157

Differentially expressed piRNA-Ls in both ADC and SCC cell lines are color with blue for down regulated and brown for upregulated in these NSCLC cell lines.
