# Supplementary material for: A piRNA-like small RNA interacts with and modulates p-ERM proteins in human somatic cells
Source: Nat Commun. 2015 Jun 22;6:7316. doi: 10.1038/ncomms8316 (PMC4557300; doi:10.1038/ncomms8316)
Supplement: Supplementary Data 1 — piRNAs matched in NCBI database [file ncomms8316-s2.doc]

**Supplementary Data 1.** piRNAs matched in the NCBI database

piRNA_known_1 DQ584518.1_piR-51630_1

piRNA_known_2 DQ584518.1_piR-51630_2

piRNA_known_3 DQ590548.1_piR-57660_1,DQ597916.1_piR-35982_1

piRNA_known_4 DQ571812.1_piR-31924_1,DQ595533.1_piR-61645_1,DQ590548.1_piR-57660_2

piRNA_known_5 DQ598378.1_piR-36444_1

piRNA_known_6 DQ595292.1_piR-61404_1

piRNA_known_7 DQ598409.1_piR-36475_5

piRNA_known_8 DQ600952.1_piR-39018_1,DQ600951.1_piR-39017_1

piRNA_known_9 DQ593356.1_piR-33468_1,DQ597347.1_piR-35413_1,DQ593049.1_piR-33161_1,DQ597346.1_piR-35412_1,DQ597345.1_piR-35411_1,DQ597344.1_piR-35410_1

piRNA_known_10 DQ594453.1_piR-60565_1

piRNA_known_11 DQ576872.1_piR-44984_1

piRNA_known_12 DQ576872.1_piR-44984_2

piRNA_known_13 DQ593358.1_piR-33470_1,DQ598639.1_piR-36705_2

piRNA_known_14 DQ599117.1_piR-37183_2

piRNA_known_15 DQ598639.1_piR-36705_3,DQ599117.1_piR-37183_3,DQ592148.1_piR-59260_2,DQ599116.1_piR-37182_2,DQ592147.1_piR-59259_2

piRNA_known_16 DQ598639.1_piR-36705_4,DQ599117.1_piR-37183_4,DQ592148.1_piR-59260_3,DQ599116.1_piR-37182_3,DQ592147.1_piR-59259_3

piRNA_known_17 DQ598639.1_piR-36705_5,DQ592148.1_piR-59260_4,DQ592147.1_piR-59259_4

piRNA_known_18 DQ575882.1_piR-43994_2,DQ594465.1_piR-60577_2,DQ575881.1_piR-43993_2,DQ594464.1_piR-60576_2,DQ593408.1_piR-33520_2,DQ593052.1_piR-33164_2

piRNA_known_19 DQ575882.1_piR-43994_3,DQ594465.1_piR-60577_3,DQ575881.1_piR-43993_3

piRNA_known_20 DQ597109.1_piR-35175_1,DQ597110.1_piR-35176_1

piRNA_known_21 DQ593325.1_piR-33437_1,DQ593431.1_piR-33543_1

piRNA_known_22 DQ575882.1_piR-43994_4,DQ594465.1_piR-60577_4,DQ594464.1_piR-60576_3

piRNA_known_23 DQ592148.1_piR-59260_5,DQ592147.1_piR-59259_5,DQ593358.1_piR-33470_2

piRNA_known_24 DQ595536.1_piR-61648_1,DQ595535.1_piR-61647_1,DQ582567.1_piR-32679_1,DQ570956.1_piR-31068_1

piRNA_known_25 DQ593358.1_piR-33470_3

piRNA_known_26 DQ598409.1_piR-36475_2

piRNA_known_27 DQ571003.1_piR-31115_1

piRNA_known_28 DQ597218.1_piR-35284_1

piRNA_known_29 DQ597997.1_piR-36063_2

piRNA_known_30 DQ594453.1_piR-60565_2

piRNA_known_31 DQ598639.1_piR-36705_1,DQ599117.1_piR-37183_1,DQ599116.1_piR-37182_1,DQ592148.1_piR-59260_1,DQ592147.1_piR-59259_1

piRNA_known_32 DQ593158.1_piR-33270_1

piRNA_known_33 DQ597971.1_piR-36037_1

piRNA_known_34 DQ598409.1_piR-36475_1

piRNA_known_35 DQ599371.1_piR-37437_1

piRNA_known_36 DQ571589.1_piR-31701_1

piRNA_known_37 DQ597482.1_piR-35548_1

piRNA_known_38 DQ582264.1_piR-32376_1

piRNA_known_39 DQ571526.1_piR-31638_1,DQ571525.1_piR-31637_1

piRNA_known_40 DQ575882.1_piR-43994_1,DQ594465.1_piR-60577_1,DQ575881.1_piR-43993_1,DQ593052.1_piR-33164_1,DQ594464.1_piR-60576_1,DQ593408.1_piR-33520_1

piRNA_known_41 DQ580405.1_piR-48517_1,DQ596542.1_piR-34608_1

piRNA_known_42 DQ594556.1_piR-60668_1

piRNA_known_43 DQ598008.1_piR-36074_1

piRNA_known_44 DQ597997.1_piR-36063_1

piRNA_known_45 DQ596183.1_piR-34249_1

piRNA_known_46 DQ571873.1_piR-31985_1

piRNA_known_47 DQ597972.1_piR-36038_1,DQ597974.1_piR-36040_1,DQ597975.1_piR-36041_1,DQ597973.1_piR-36039_1

piRNA_known_48 DQ580006.1_piR-48118_1

piRNA_known_49 DQ597215.1_piR-35281_1

piRNA_known_50 DQ571003.1_piR-31115_2

piRNA_known_51 DQ598159.1_piR-36225_2

piRNA_known_52 DQ594740.1_piR-60852_1

piRNA_known_53 DQ570968.1_piR-31080_1

piRNA_known_54 DQ598639.1_piR-36705_6,DQ592148.1_piR-59260_6,DQ592147.1_piR-59259_6

piRNA_known_55 DQ597216.1_piR-35282_2

piRNA_known_56 DQ599457.1_piR-37523_1,DQ599459.1_piR-37525_1,DQ599458.1_piR-37524_1

piRNA_known_57 DQ598409.1_piR-36475_3

piRNA_known_58 DQ576880.1_piR-44992_1

piRNA_known_59 DQ576605.1_piR-44717_1,DQ593909.1_piR-34021_1

piRNA_known_60 DQ576605.1_piR-44717_2

piRNA_known_61 DQ576880.1_piR-44992_2

piRNA_known_62 DQ576605.1_piR-44717_3

piRNA_known_63 DQ592932.1_piR-33044_1

piRNA_known_64 DQ573323.1_piR-41435_1

piRNA_known_65 DQ577008.1_piR-45120_1

piRNA_known_66 DQ590835.1_piR-57947_1

piRNA_known_67 DQ577008.1_piR-45120_2

piRNA_known_68 DQ590835.1_piR-57947_2

piRNA_known_69 DQ597217.1_piR-35283_2,DQ597216.1_piR-35282_3

piRNA_known_70 DQ596310.1_piR-34376_1,DQ596311.1_piR-34377_1,DQ570393.1_piR-30505_1,DQ570394.1_piR-30506_1

piRNA_known_71 DQ597971.1_piR-36037_2

piRNA_known_72 DQ595023.1_piR-61135_1

piRNA_known_73 DQ598651.1_piR-36717_1

piRNA_known_74 DQ576872.1_piR-44984_3

piRNA_known_75 DQ576872.1_piR-44984_4

piRNA_known_76 DQ576872.1_piR-44984_5

piRNA_known_77 DQ598409.1_piR-36475_4

piRNA_known_78 DQ598180.1_piR-36246_1

piRNA_known_79 DQ587229.1_piR-54341_1

piRNA_known_80 DQ597347.1_piR-35413_2

piRNA_known_81 DQ597346.1_piR-35412_2,DQ597347.1_piR-35413_3,DQ597344.1_piR-35410_2,DQ597345.1_piR-35411_2,DQ593049.1_piR-33161_2,DQ593356.1_piR-33468_2

piRNA_known_82 DQ600952.1_piR-39018_2,DQ600951.1_piR-39017_2

piRNA_known_83 DQ571003.1_piR-31115_3

piRNA_known_84 DQ580006.1_piR-48118_2

piRNA_known_85 DQ571549.1_piR-31661_1,DQ571550.1_piR-31662_1

piRNA_known_86 DQ595536.1_piR-61648_2,DQ595535.1_piR-61647_2,DQ571813.1_piR-31925_1,DQ595534.1_piR-61646_1,DQ595533.1_piR-61645_2,DQ571812.1_piR-31924_2,DQ570956.1_piR-31068_2,DQ595532.1_piR-61644_1,DQ582567.1_piR-32679_2

piRNA_known_87 DQ595536.1_piR-61648_3,DQ595535.1_piR-61647_3,DQ590386.1_piR-57498_1,DQ595534.1_piR-61646_2,DQ571813.1_piR-31925_2,DQ595533.1_piR-61645_3,DQ571812.1_piR-31924_3,DQ570956.1_piR-31068_3,DQ595532.1_piR-61644_2,DQ582567.1_piR-32679_3

piRNA_known_88 DQ570956.1_piR-31068_4,DQ582567.1_piR-32679_4,DQ571813.1_piR-31925_3,DQ571812.1_piR-31924_4,DQ595536.1_piR-61648_4

piRNA_known_89 DQ597916.1_piR-35982_2,DQ582567.1_piR-32679_5,DQ571812.1_piR-31924_5,DQ571813.1_piR-31925_4,DQ595536.1_piR-61648_5,DQ595533.1_piR-61645_4,DQ595534.1_piR-61646_3,DQ595535.1_piR-61647_4,DQ595532.1_piR-61644_3

piRNA_known_90 DQ572892.1_piR-41004_1

piRNA_known_91 DQ584518.1_piR-51630_3

piRNA_known_92 DQ598312.1_piR-36378_2

piRNA_known_93 DQ582567.1_piR-32679_6,DQ571812.1_piR-31924_6,DQ571813.1_piR-31925_5,DQ595536.1_piR-61648_6

piRNA_known_94 DQ571511.1_piR-31623_1

piRNA_known_95 DQ570940.1_piR-31052_1

piRNA_known_96 DQ570698.1_piR-30810_1

piRNA_known_97 DQ571858.1_piR-31970_1

piRNA_known_98 DQ598639.1_piR-36705_7,DQ599117.1_piR-37183_5,DQ592148.1_piR-59260_7,DQ599116.1_piR-37182_4,DQ592147.1_piR-59259_7

piRNA_known_99 DQ598183.1_piR-36249_1,DQ598181.1_piR-36247_1,DQ598182.1_piR-36248_1

piRNA_known_100 DQ598183.1_piR-36249_2,DQ598182.1_piR-36248_2,DQ598181.1_piR-36247_2

piRNA_known_101 DQ598183.1_piR-36249_3,DQ598182.1_piR-36248_3,DQ598181.1_piR-36247_3

piRNA_known_102 DQ598183.1_piR-36249_4,DQ598182.1_piR-36248_4,DQ598181.1_piR-36247_4

piRNA_known_103 DQ570999.1_piR-31111_1

piRNA_known_104 DQ570968.1_piR-31080_2

piRNA_known_105 DQ585304.1_piR-52416_1,DQ585303.1_piR-52415_1,DQ585302.1_piR-52414_1

piRNA_known_106 DQ595186.1_piR-61298_1

piRNA_known_107 DQ593767.1_piR-33879_1

piRNA_known_108 DQ593358.1_piR-33470_4,DQ598639.1_piR-36705_8

piRNA_known_109 DQ599117.1_piR-37183_6

piRNA_known_110 DQ571549.1_piR-31661_2,DQ571550.1_piR-31662_2,DQ582231.1_piR-32343_1

piRNA_known_111 DQ598183.1_piR-36249_5,DQ598182.1_piR-36248_5,DQ598181.1_piR-36247_5

piRNA_known_112 DQ597945.1_piR-36011_1

piRNA_known_113 DQ597945.1_piR-36011_2

piRNA_known_114 DQ597945.1_piR-36011_3

piRNA_known_115 DQ597945.1_piR-36011_4

piRNA_known_116 DQ593415.1_piR-33527_1,DQ593414.1_piR-33526_1

piRNA_known_117 DQ597945.1_piR-36011_5

piRNA_known_118 DQ597945.1_piR-36011_6

piRNA_known_119 DQ597945.1_piR-36011_7

piRNA_known_120 DQ597945.1_piR-36011_8

piRNA_known_121 DQ597945.1_piR-36011_9

piRNA_known_122 DQ597945.1_piR-36011_10

piRNA_known_123 DQ590835.1_piR-57947_3

piRNA_known_124 DQ576605.1_piR-44717_4,DQ593909.1_piR-34021_2

piRNA_known_125 DQ598175.1_piR-36241_1

piRNA_known_126 DQ575884.1_piR-43996_1,DQ575883.1_piR-43995_1,DQ575881.1_piR-43993_4,DQ594464.1_piR-60576_4,DQ593408.1_piR-33520_3,DQ593052.1_piR-33164_3

piRNA_known_127 DQ597971.1_piR-36037_3

piRNA_known_128 DQ571524.1_piR-31636_1

piRNA_known_129 DQ571526.1_piR-31638_2

piRNA_known_130 DQ598675.1_piR-36741_1

piRNA_known_131 DQ595536.1_piR-61648_7,DQ571813.1_piR-31925_6,DQ571812.1_piR-31924_7,DQ582567.1_piR-32679_7,DQ570956.1_piR-31068_5

piRNA_known_132 DQ592953.1_piR-33065_1

piRNA_known_133 DQ571500.1_piR-31612_1

piRNA_known_134 DQ576872.1_piR-44984_6

piRNA_known_135 DQ598263.1_piR-36329_1

piRNA_known_136 DQ571333.1_piR-31445_1

piRNA_known_137 DQ587269.1_piR-54381_1

piRNA_known_138 DQ588101.1_piR-55213_1

piRNA_known_139 DQ598294.1_piR-36360_1

piRNA_known_140 DQ596993.1_piR-35059_1

piRNA_known_141 DQ572870.1_piR-40982_1,DQ581967.1_piR-32079_1,DQ571849.1_piR-31961_1,DQ593538.1_piR-33650_1

piRNA_known_142 DQ581033.1_piR-49145_1

piRNA_known_143 DQ598650.1_piR-36716_1,DQ598649.1_piR-36715_1,DQ598648.1_piR-36714_1,DQ598647.1_piR-36713_1

piRNA_known_144 DQ570687.1_piR-30799_1

piRNA_known_145 DQ597916.1_piR-35982_3,DQ582567.1_piR-32679_8,DQ571812.1_piR-31924_8,DQ595532.1_piR-61644_4,DQ595533.1_piR-61645_5

piRNA_known_146 DQ597482.1_piR-35548_3

piRNA_known_147 DQ594453.1_piR-60565_3,DQ575659.1_piR-43771_1,DQ575660.1_piR-43772_1

piRNA_known_148 DQ597482.1_piR-35548_2

piRNA_known_149 DQ594464.1_piR-60576_5,DQ575881.1_piR-43993_5,DQ593052.1_piR-33164_4,DQ593408.1_piR-33520_4

piRNA_known_150 DQ571524.1_piR-31636_2

piRNA_known_151 DQ571524.1_piR-31636_3

piRNA_known_152 DQ595532.1_piR-61644_5,DQ595533.1_piR-61645_6

piRNA_known_153 DQ598183.1_piR-36249_6,DQ598182.1_piR-36248_6,DQ598181.1_piR-36247_6

piRNA_known_154 DQ570812.1_piR-30924_1

piRNA_known_155 DQ595536.1_piR-61648_8,DQ595535.1_piR-61647_5,DQ595534.1_piR-61646_4,DQ590386.1_piR-57498_2,DQ571813.1_piR-31925_7,DQ570956.1_piR-31068_6

piRNA_known_156 DQ582536.1_piR-32648_1

piRNA_known_157 DQ590013.1_piR-57125_1

piRNA_known_158 DQ570728.1_piR-30840_1

piRNA_known_159 DQ575658.1_piR-43770_1

piRNA_known_160 DQ594465.1_piR-60577_5,DQ575881.1_piR-43993_6,DQ575882.1_piR-43994_5

piRNA_known_161 DQ575882.1_piR-43994_6,DQ594465.1_piR-60577_6,DQ575881.1_piR-43993_7,DQ593052.1_piR-33164_5,DQ594464.1_piR-60576_6,DQ593408.1_piR-33520_5

piRNA_known_162 DQ594464.1_piR-60576_7,DQ575881.1_piR-43993_8,DQ593052.1_piR-33164_6,DQ593408.1_piR-33520_6

piRNA_known_163 DQ594464.1_piR-60576_8,DQ575881.1_piR-43993_9,DQ593052.1_piR-33164_7,DQ593408.1_piR-33520_7

piRNA_known_164 DQ594464.1_piR-60576_9,DQ594465.1_piR-60577_7,DQ575882.1_piR-43994_7

piRNA_known_165 DQ593407.1_piR-33519_1,DQ598677.1_piR-36743_1,DQ598676.1_piR-36742_1

piRNA_known_166 DQ576880.1_piR-44992_3

piRNA_known_167 DQ598159.1_piR-36225_3

piRNA_known_168 DQ597346.1_piR-35412_3,DQ597347.1_piR-35413_4,DQ597345.1_piR-35411_3,DQ593049.1_piR-33161_3,DQ593356.1_piR-33468_3

piRNA_known_169 DQ600952.1_piR-39018_3,DQ600951.1_piR-39017_3

piRNA_known_170 DQ575881.1_piR-43993_10,DQ593052.1_piR-33164_8,DQ593408.1_piR-33520_8,DQ594464.1_piR-60576_10

piRNA_known_171 DQ600952.1_piR-39018_4,DQ600951.1_piR-39017_4

piRNA_known_172 DQ593356.1_piR-33468_4,DQ597347.1_piR-35413_5,DQ597346.1_piR-35412_4,DQ597345.1_piR-35411_4,DQ597344.1_piR-35410_3

piRNA_known_173 DQ575882.1_piR-43994_8,DQ594465.1_piR-60577_8,DQ575881.1_piR-43993_11,DQ594464.1_piR-60576_11,DQ593408.1_piR-33520_9,DQ593052.1_piR-33164_9

piRNA_known_174 DQ593736.1_piR-33848_1

piRNA_known_175 DQ593039.1_piR-33151_1

piRNA_known_176 DQ590835.1_piR-57947_6

piRNA_known_177 DQ597108.1_piR-35174_1,DQ597110.1_piR-35176_3,DQ597109.1_piR-35175_2

piRNA_known_178 DQ593431.1_piR-33543_3

piRNA_known_179 DQ590830.1_piR-57942_1

piRNA_known_180 DQ573323.1_piR-41435_2

piRNA_known_181 DQ594465.1_piR-60577_9,DQ575881.1_piR-43993_12,DQ575882.1_piR-43994_9

piRNA_known_182 DQ598159.1_piR-36225_4

piRNA_known_183 DQ597971.1_piR-36037_4

piRNA_known_184 DQ570991.1_piR-31103_1,DQ570992.1_piR-31104_1,DQ597347.1_piR-35413_6,DQ593356.1_piR-33468_5

piRNA_known_185 DQ600951.1_piR-39017_5,DQ600952.1_piR-39018_5

piRNA_known_186 DQ576603.1_piR-44715_1,DQ576604.1_piR-44716_1

piRNA_known_187 DQ576603.1_piR-44715_2,DQ576604.1_piR-44716_2

piRNA_known_188 DQ576917.1_piR-45029_1

piRNA_known_189 DQ597971.1_piR-36037_5

piRNA_known_190 DQ597971.1_piR-36037_6

piRNA_known_191 DQ597971.1_piR-36037_7

piRNA_known_192 DQ597403.1_piR-35469_1,DQ597402.1_piR-35468_1,DQ597401.1_piR-35467_1

piRNA_known_193 DQ598312.1_piR-36378_3

piRNA_known_194 DQ575881.1_piR-43993_13,DQ593052.1_piR-33164_10,DQ593408.1_piR-33520_10,DQ594464.1_piR-60576_12

piRNA_known_195 DQ597971.1_piR-36037_8

piRNA_known_196 DQ575881.1_piR-43993_14,DQ593408.1_piR-33520_11,DQ594464.1_piR-60576_13,DQ593052.1_piR-33164_11

piRNA_known_197 DQ597110.1_piR-35176_8

piRNA_known_198 DQ593431.1_piR-33543_8

piRNA_known_199 DQ597960.1_piR-36026_1

piRNA_known_200 DQ595536.1_piR-61648_9,DQ595535.1_piR-61647_6,DQ582567.1_piR-32679_9,DQ597916.1_piR-35982_4

piRNA_known_201 DQ598190.1_piR-36256_1

piRNA_known_202 DQ598190.1_piR-36256_2

piRNA_known_203 DQ598187.1_piR-36253_1,DQ598189.1_piR-36255_1,DQ598188.1_piR-36254_1,DQ598190.1_piR-36256_3

piRNA_known_204 DQ598187.1_piR-36253_2,DQ598188.1_piR-36254_2,DQ598189.1_piR-36255_2,DQ598190.1_piR-36256_4,DQ598619.1_piR-36685_1

piRNA_known_205 DQ587229.1_piR-54341_2

piRNA_known_206 DQ598190.1_piR-36256_5,DQ598189.1_piR-36255_3

piRNA_known_207 DQ598190.1_piR-36256_6,DQ598189.1_piR-36255_4,DQ598177.1_piR-36243_1,DQ598188.1_piR-36254_3,DQ598176.1_piR-36242_1,DQ598187.1_piR-36253_3

piRNA_known_208 DQ587229.1_piR-54341_3

piRNA_known_209 DQ594453.1_piR-60565_5

piRNA_known_210 DQ598951.1_piR-37017_1

piRNA_known_211 DQ592931.1_piR-33043_1

piRNA_known_212 DQ594453.1_piR-60565_4

piRNA_known_213 DQ597217.1_piR-35283_4,DQ597216.1_piR-35282_5

piRNA_known_214 DQ580006.1_piR-48118_3

piRNA_known_215 DQ598951.1_piR-37017_2

piRNA_known_216 DQ598409.1_piR-36475_6

piRNA_known_217 DQ584518.1_piR-51630_5

piRNA_known_218 DQ584518.1_piR-51630_6

piRNA_known_219 DQ584518.1_piR-51630_7

piRNA_known_220 DQ597997.1_piR-36063_3

piRNA_known_221 DQ571003.1_piR-31115_4

piRNA_known_222 DQ584518.1_piR-51630_4

piRNA_known_223 DQ571524.1_piR-31636_4

piRNA_known_224 DQ597484.1_piR-35550_1,DQ597483.1_piR-35549_1

piRNA_known_225 DQ588040.1_piR-55152_1

piRNA_known_226 DQ576605.1_piR-44717_5,DQ593909.1_piR-34021_3

piRNA_known_227 DQ576605.1_piR-44717_6,DQ593909.1_piR-34021_4

piRNA_known_228 DQ584698.1_piR-51810_1

piRNA_known_229 DQ579049.1_piR-47161_1

piRNA_known_230 DQ571524.1_piR-31636_6

piRNA_known_231 DQ590835.1_piR-57947_7

piRNA_known_232 DQ577008.1_piR-45120_3

piRNA_known_233 DQ571524.1_piR-31636_5

piRNA_known_234 DQ581032.1_piR-49144_1

piRNA_known_235 DQ598409.1_piR-36475_7

piRNA_known_236 DQ580006.1_piR-48118_4

piRNA_known_237 DQ584197.1_piR-51309_1

piRNA_known_238 DQ598675.1_piR-36741_2

piRNA_known_239 DQ571526.1_piR-31638_3

piRNA_known_240 DQ582036.1_piR-32148_1

piRNA_known_241 DQ593292.1_piR-33404_1

piRNA_known_242 DQ576780.1_piR-44892_1

piRNA_known_243 DQ582264.1_piR-32376_2

piRNA_known_244 DQ597483.1_piR-35549_2,DQ597484.1_piR-35550_2

piRNA_known_245 DQ597482.1_piR-35548_4

piRNA_known_246 DQ597990.1_piR-36056_1

piRNA_known_247 DQ571875.1_piR-31987_1,DQ571874.1_piR-31986_1

piRNA_known_248 DQ596276.1_piR-34342_1

piRNA_known_249 DQ598641.1_piR-36707_1

piRNA_known_250 DQ596738.1_piR-34804_1

piRNA_known_251 DQ600436.1_piR-38502_1

piRNA_known_252 DQ579193.1_piR-47305_1

piRNA_known_253 DQ596469.1_piR-34535_1,DQ596470.1_piR-34536_1,DQ596468.1_piR-34534_1

piRNA_known_254 DQ584698.1_piR-51810_2

piRNA_known_255 DQ571378.1_piR-31490_1

piRNA_known_256 DQ573352.1_piR-41464_1

piRNA_known_257 DQ582566.1_piR-32678_1

piRNA_known_258 DQ576200.1_piR-44312_1

piRNA_known_259 DQ576200.1_piR-44312_2

piRNA_known_260 DQ576200.1_piR-44312_3
