# Supplementary material for: A piRNA-like small RNA interacts with and modulates p-ERM proteins in human somatic cells
Source: Nat Commun. 2015 Jun 22;6:7316. doi: 10.1038/ncomms8316 (PMC4557300; doi:10.1038/ncomms8316)
Supplement: Supplementary Data 2 — Novel piRNA like small RNAs [file ncomms8316-s3.docx]

**Supplementary Data 2.** Novel piRNA like small RNAs

>piRNA-L-2_igs

UCUGCACAGCCGCUUUCCACACAGACAUC

>piRNA-L-3_igs

AGGAGUUCUGGGCUGUAGUGCGCUAUGCC

>piRNA-L-6_igs

AGACUAGUCAAGUGCAGUAGUGAGAAG

>piRNA-L-8_igs

CAGAGUGCCAACCAUUACACAAUGGAACC

>piRNA-L-9_igs

UAAAGACGUUAGGUCAAGGUGUAGCC

>piRNA-L-13_igs

AGGGUUCAGCUGUCUCUUACUUUUAACC

>piRNA-L-14_igs

GUCAGGAUGGCCGAGCGGUCUAAGGC

>piRNA-L-18_igs

UGGUUCGUCCAAGUGCACUUUCCAGU

>piRNA-L-20_igs

GGUUCCAUGGUGUAAUGGUAAGCACUCUG

>piRNA-L-21_igs

AAGCCGGCUUCACGCUCAGGAGAAAACGC

>piRNA-L-22_igs

UGCUCAGAAUUUAUUAAUUUUCACGGU

>piRNA-L-23_igs

AAAUUGUUCAAGGUCUAUCAGUGGAUGCAG

>piRNA-L-27_igs

GGCUGAUCUGGCUGGCUAGGCGGGUGUCC

>piRNA-L-30_igs

CAGGAAUCCUAACCGCUAGACCAUAUGGGA

>piRNA-L-33_igs

UUUUAUGAGUGAAACAUAAGAGUCUGACA

>piRNA-L-34_igs

GCCUGGGAAUAACGGGUGCUGUAGGCUUU

>piRNA-L-35_igs

CCUAGGGCUCAGAGCACUGCAGCAGAU

>piRNA-L-36_igs

GGUUCCAUGGUGUAAUGGUUAGCAUUCUG

>piRNA-L-37_igs

UGAGAAUUCUGCCACUGAACCACCCAUGC

>piRNA-L-40_igs

GGUUCCAUGGUGUAAUGGUGAGCACUCUG

>piRNA-L-41_igs

GCCCAGCACGCUUCCGCUGCGCCACUCUGCU

>piRNA-L-42_igs

GCGUGCCUGUAGUCCCAGCUACUCGGG

>piRNA-L-44_igs

ACCCAGGAUCUCCUGUUUACGAGACAG

>piRNA-L-45_igs

UCUGAUAACCCACUACCAUCGGACCAGCC

>piRNA-L-46_igs

CAUGCGCUCUACCACUGAGCUACAUCCCC

>piRNA-L-47_igs

UUCUCACUACUGCACUUGACUAGUCU

>piRNA-L-48_igs

CUGAUGCUCUACCAACUGAGCUAUCCAGG

>piRNA-L-49_igs

ACCUCUCAUGUCUCUUCACCAUGCCAG

>piRNA-L-50_igs

UCAGGGCCCUGUUCAACUAAGCACUCUACUC

>piRNA-L-51_igs

GUAUUAGAGGCACCGCCUGCCCAGUGA

>piRNA-L-52_igs

AUCUGUGAUGUGAACCACAACCUGAGU

>piRNA-L-55_igs

UCCUUUUUAGUAUAGUGGUGAGUAUCCCC

>piRNA-L-61_igs

CAGGGUCGGGCCUGGUUAGUACUUGGA

>piRNA-L-62_igs

UGAGCCGAGAUCACGCCACUGCACUCC

>piRNA-L-66_igs

GCCCAGCACGCUUCCGCUGCGCCACUCUGCU

>piRNA-L-67_igs

AAAGCCUACAGCACCCGGUAUUCCCAGG

>piRNA-L-70_igs

AGACUAGUCAAGUGCAGUAGUGAGAAG

>piRNA-L-71_igs

GGCUAAGAUCAAGUGUAGUAUCUGUU

>piRNA-L-72_igs

GUCAGGAUGGCCGAGCGGUCUAAGGC

>piRNA-L-73_igs

CGGACAGGAUUGACAGAUUGAUAGCU

>piRNA-L-75_igs

GCAUGAGUGGUUCUGUGGUAGAAUUCUC

>piRNA-L-79_igs

UGAUCAGAGCCCAGUGCUGGACAUCAUGGGA

>piRNA-L-81_igs

GUCAGGAUGGCCGAGCGGUCUAAGGC

>piRNA-L-82_igs

CCUGGACUCAAGCGAUCCUCCAGCCUCAGCCU

>piRNA-L-85_igs

UGGCUGAUUUGCGUUCAGUUGAUGCAGA

>piRNA-L-87_igs

CAGAGUGCUCACCAUUACACCAUGGAACC

>piRNA-L-88_igs

GCGUUGGUGGUAUAGUGGUGAGCAUAGCUGC

>piRNA-L-89_igs

GGGUCUUAGCUAUUGUGUGUUCAGAUAUGU

>piRNA-L-91_igs

ACCACUCAGACCGCGUUCUCUCCCUC

>piRNA-L-94_igs

GGUUCCAUGGUGUAAUGGUUAGCACUCUG

>piRNA-L-95_igs

GCAUUGGCGGUACAGUGGCAGAAUUCUCG

>piRNA-L-96_igs

GUCCUGCAAUUCACAUUAAUUCUCACAGCU

>piRNA-L-97_igs

UGGGUCUUAGCUAUUGUGUGUUCAGA

>piRNA-L-98_igs

CAGUGCUCUAACCCCUGAGCUAUGGAGCC

>piRNA-L-99_igs

UUGAGAUACUGACUAGUCUGGUGUUAUUU

>piRNA-L-100_igs

UAGUGGGUUAUCAGAACUUAUUAACA

>piRNA-L-101_igs

UAACAGGGGCCCUCUCAGCCCUCCUAAUG

>piRNA-L-106_igs

UACCUGAUGCAUGAUCUCUACAGUUCUGAGA

>piRNA-L-107_igs

CAAGAAUUCUACCACUGAACAACCAAUGC

>piRNA-L-109_igs

UGGCUUUAGCUCAGCGGUUACUUCGAC

>piRNA-L-110_igs

AGGGUGGUUCAGUGGUAGAAUUCUCG

>piRNA-L-111_igs

CUUAAUGAUGACUGUUUUUUUUGAUUGCU

>piRNA-L-112_igs

AGCCUGGUGAUAGCUGGUUGUCCAAG

>piRNA-L-113_igs

GUUUGGAGGUUCUAGCAGGGGAGCGCA

>piRNA-L-114_igs

AGUGACGCUCAGACAGGCAUAGCCCUGGGAG

>piRNA-L-119_igs

AGACUAGUCAAGUGCAGUAGUGAGAAG

>piRNA-L-121_igs

UCUGAAACCAAUUUUUUGAGGCCUUGCGU

>piRNA-L-123_igs

CAGAGUGCUAACCAUUGCACCAUAGAACC

>piRNA-L-124_igs

AGCCUGGUGAUAGCUGGUUGUCCAAGAU

>piRNA-L-127_igs

UCCUUGGUGGUCUAGUGGUUAGGAUUCGGCU

>piRNA-L-128_igs

CAGAUUCACAAUCUGAUGUUUUGGUUAAACU

>piRNA-L-129_igs

CAUGAAUGGAUGAACGAGAUUCCCACU

>piRNA-L-130_igs

CAGAGUGCUCACCAUUACACCAUGGAACC

>piRNA-L-132_igs

AGUUGUAAACACCACUGCACUCGGACCAGCC

>piRNA-L-133_igs

AGUUGUAAACACCACUGCACUCGGACCAGCC

>piRNA-L-136_igs

GGUAGUGUGGCCGAGCGGUCUAAGGC

>piRNA-L-138_igs

ACUUUAGCUCUAGAAUUACUCUGAGACCU

>piRNA-L-139_igs

UUCUCACUACUGCACUUGACUAGUCU

>piRNA-L-141_igs

CUGAAAUGAAGAGAAUACUCAUUGCUGAUC

>piRNA-L-142_igs

ACCACUCAGACCGCGUUCUCUCCCUC

>piRNA-L-143_igs

UUUCCUAGUGUCCAAAGAGCUGUUCCU

>piRNA-L-144_igs

UCAGAACUUCCACAAAAUCAUUUGUU

>piRNA-L-145_igs

AGCCAGCCAGAUCAGCCGAAUCAACCC

>piRNA-L-146_igs

AGACUAGUCAAGUGCAGUAGUGAGAA

>piRNA-L-149_igs

UGAGCCGAGAUCACGCCACUGCACUCC

>piRNA-L-152_igs

AGACUAGUCAAGUGCAGUAGUGAGAAG

>piRNA-L-155_igs

ACAAUUCCUUGGCUGUGUCUGAGCAC

>piRNA-L-156_igs

GCAUUGGUGGUUCAGUAGUAGAAUUCUCG

>piRNA-L-160_igs

CAUUUGGCAGAAUCAUUACAUCAUUGGUU

>piRNA-L-161_igs

CGGGUCUUCCAGGAGUCGGGUUGCUU

>piRNA-L-163_igs

AUAUCAUGAUGUUACUUUGAUUCUCUGACC

>piRNA-L-169_igs

UGGUUCCCUGACCGGGAAUUGAACCC

>piRNA-L-170_igs

GUUAAUAAGUUCUGAUAACCCACUAC

>piRNA-L-171_igs

AAGCCAGUCAAAUUUAGCAGUGGGGGG

>piRNA-L-174_igs

GCGUUGGUGGUAUAGUGGUGAGCAUAGCUGC

>piRNA-L-177_igs

GAUGAUGAUGAGAAGCCUUUUGCUGAGC

>piRNA-L-179_igs

GCUCUACACGUUCAGAGAAACUUCUCU

>piRNA-L-180_igs

CCUCAGACGCAGGCUUUCUUCAGUAGA

>piRNA-L-181_igs

UAAAACUCAAAGGACCUGGCGGUGCUU

>piRNA-L-182_igs

CCUGGAUAGCUCAGUUGGUAGAGCAUCAG

>piRNA-L-183_igs

GCAUGGGUCGUUCAGUGGUAGAAUUCUCU

>piRNA-L-184_igs

AGCCUGGUGAUAGCUGGUUGUCCAAGAUA

>piRNA-L-188_igs

AAGUCCUACGUGAUCUGAGUUCAGACCG

>piRNA-L-189_igs

GUCAGGAUGGCCGAGCGGUCUAAGGC

>piRNA-L-190_igs

AGGAGUUCUGGGCUGUAGUGCGCUAUGC

>piRNA-L-191_igs

ACCCAGGAUCUCCUGUUUACAAGACAG

>piRNA-L-192_igs

AGCUGCGCUCCCCUGCUAGAACCUCC

>piRNA-L-193_igs

CAGGGUCGGGCCUGGUUAGUACUUGGA

>piRNA-L-194_igs

GGUUCCAUGGUGUAAUGGUAAGCACUCUG

>piRNA-L-196_igs

GAGUCAGCUAAAUACUUUGACGCCGGU

>piRNA-L-198_igs

UUGGUGGUAUAGUGGUGAGCAUAGCUG

>piRNA-L-199_igs

AGGCCAUGAUGACAGUAUUUCUGAGU

>piRNA-L-201_igs

AAAGCCUACAGCACCCGGUAUUCCCAGG

>piRNA-L-202_igs

CAUGCGCUCUACCACUGAGCUACAUCCCC

>piRNA-L-203_igs

AUCUGUGAUGAUAGAAAUUGGAUCUGAGG

>piRNA-L-205_igs

GGCUGGUCCGAUGGUAGCAGGUUAUCAGAACU

>piRNA-L-207_igs

UCCAAGUACUAACCAGGCCCGACCCUGC

>piRNA-L-208_igs

AGUCUCAGUUUCCUCUGCAAACAGUU

>piRNA-L-210_igs

UGUCACCCCAUUGAUCGCCAGGGUUGAUU

>piRNA-L-211_igs

GGUCAAUGAUGAAUGGUAAAAGGUCUGAGU

>piRNA-L-212_igs

UAUCUGAACACACAAUAGCUAAGACCC

>piRNA-L-214_igs

AUCCAGCGGUUGUCAGCUAUCCAGGCU

>piRNA-L-216_igs

GAGCGCUCUACCGCCUGAGCUAAUUCCCC

>piRNA-L-218_igs

AGGAGUUCUGGGCUGUAGUGCGCUAUGCC

>piRNA-L-219_igs

CAUGAAUGGAUGAACGAGAUUCCCACU

>piRNA-L-221_igs

CACCAGUGUGAGUUCUACCAUUGCCAAA

>piRNA-L-225_igs

UCACAGUGAAUUCUACCAGUGCCAUA

>piRNA-L-227_igs

GUGGGCCACUUUUGGUAAGCAGAACUG

>piRNA-L-228_igs

UCAUGCUCUAUCGACUGAGCUAGCCGGG

>piRNA-L-230_igs

AGACUAGUCAAGUGCAGUAGUGAGAAG

>piRNA-L-232_igs

GGCUGGUCCGAGUGCAGUGGUGUUUACAACU

>piRNA-L-234_igs

AGACUAGUCAAGUGCAGUAGUGAGAAG

>piRNA-L-236_igs

UAGUGGGUUAUCAGAACUUAUUAACA

>piRNA-L-237_igs

UCUCAGAACUGUAGAGAUCAUGCAUCAGGUA

>piRNA-L-241_igs

AGACUAGUCAAGUGCAGUAGUGAGAAG

>piRNA-L-244_igs

UACAGUCCAAUGCUUCACUCAGCCAUUUUACC

>piRNA-L-245_igs

UGAGCCGAGAUCACGCCACUGCACUCC

>piRNA-L-246_igs

UGCUGCAGUUAAAAAGCUCCUAGUUGGAUCU

>piRNA-L-247_igs

UCUGGUGAUGAAAUGGAACGUUUCUGAUG

>piRNA-L-248_igs

AAGCCAGUCAAAUUUAGCAGUGGGGGG

>piRNA-L-252_igs

AAGUUUCAGCUUUGCAACCAUACUCCCCUCG

>piRNA-L-257_igs

UGUUGUCAGAUUCACAAUCUGAUGUUUU

>piRNA-L-260_igs

GUCAGGAUGGCCGAGCGGUCUAAGGC

>piRNA-L-261_igs

AGUUGUAAACACCACUGCACUCGGACCAGCC

>piRNA-L-264_igs

GCCCAGCACGCUUCCGCUGCGCCACUCUGCU

>piRNA-L-265_igs

ACCCAGGAUCUCCUGUUUACGAGACAG

>piRNA-L-266_igs

AAGGAUGAUGAGACAGGCUAUGCUGAAG

>piRNA-L-267_igs

UUGGUGGUAUAGUGGUGAGCAUAGCUG

>piRNA-L-269_igs

GUCAGGAUGGCCGAGCGGUCUAAGGC

>piRNA-L-270_igs

AACAUUCAUUGCUGUCGGUGGGUUUG

>piRNA-L-272_igs

AGACUAGUCAAGUGCAGUAGUGAGAAG

>piRNA-L-274_igs

GCGUUGGUGGUAUAGUGGUGAGCAUAGCUGC

>piRNA-L-276_igs

ACUUUAGCUCUAGAAUUACUCUGAGACCU

>piRNA-L-277_igs

UGCCAUCAGAACUCUAACAUGCUAUU

>piRNA-L-279_igs

UUCCAUCAGCUUUCUUUGCCAUCAUUUGGA

>piRNA-L-280_igs

UCCAAGUACUAACCAGGCCCGACCCUGCUU

>piRNA-L-281_igs

AGGCAUGAUGACUCCAACUGUGGAGACUGACU

>piRNA-L-284_igs

GCCCAGCACGCUUCCGCUGCGCCACUCUGCU

>piRNA-L-286_igs

GCUUUGGUGGUUCAGUGGUAGAAUUCUC

>piRNA-L-288_igs

AGACUAGUCAAGUGCAGUAGUGAGAAG

>piRNA-L-290_igs

UAGAGGAGCCUGUUCUGUAAUCGAUAAACC

>piRNA-L-293_igs

AACGAGACUCUGGCAUGCUAACUAGUU

>piRNA-L-294_igs

GCUCUACACGUUCAGAGAAACUUCUCU

>piRNA-L-295_igs

GUCACUCAGACAUCCAAGGAAGGUAG

>piRNA-L-296_igs

UUCUCACUACUGCACUUGACUAGUCU

>piRNA-L-298_igs

UGUCAGAAGUCAAAGCAAUUCAUCACAGACU

>piRNA-L-299_igs

UGUAAACAUCCUUGACUGGAAGCUGU

>piRNA-L-300_igs

AAGCCGGCUUCACGCUCAGGAGAAAACGC

>piRNA-L-304_igs

CUGGAGUGCAGUGGCGUGAUCUCGGCUCA

>piRNA-L-308_igs

UUGGCAGUUCAGUGGUAGAAUUCUCGC

>piRNA-L-309_igs

AUAAAUGAUGAUCAGCAGAAUCUGAGU

>piRNA-L-313_igs

UCCCUGGUGGUCUAGUGGCUAGGAUU

>piRNA-L-315_igs

UGACAUGAUGAGAUUUCACUCUGACA

>piRNA-L-316_igs

CAGGGCACGUGUUAGGACCUGAAAGA

>piRNA-L-318_igs

UGCGCUCCCCUGCUAGAACCUCCAAAC

>piRNA-L-322_igs

CAGUGCUCUAACCCCUGAGCUAUGGAGCC

>piRNA-L-323_igs

GUCGAUGAUGAUUGGUAAAAGGUCUGAUU

>piRNA-L-326_igs

GCUGACACGCUGUCCUCUGGCGACCU

>piRNA-L-329_igs

GUUUGGAGGUUCUAGCAGGGGAGCGC

>piRNA-L-331_igs

GUCAGGAUGGCCGAGCGGUCUAAGGC

>piRNA-L-334_igs

CCCGAGUAGCUGGGACUACAGGCACGC

>piRNA-L-341_igs

UCGACUUCCAUGGCCACCGUCCUGCU

>piRNA-L-342_igs

GCGUUGGUGGUAUAGUGGUGAGCAUAGCUGC

>piRNA-L-343_igs

CACUUUGACAUUCAGAGCACUGGGCAGAA

>piRNA-L-344_igs

AAAGUGAAUGAUUAAAGGUCUUGGGGCC

>piRNA-L-346_igs

AGACUAGUCAAGUGCAGUAGUGAGAAG

>piRNA-L-348_igs

GUUCACCAUCUUUGGGGUCCUAACACG

>piRNA-L-350_igs

UAUCUGAACACACAAUAGCUAAGACCC

>piRNA-L-351_igs

AGACUAGUCAAGUGCAGUAGUGAGAAG

>piRNA-L-352_igs

UGGGUUCGAAUCCCAUCCUCGUCGGCC

>piRNA-L-354_igs

CCGGGGACCUCUUGAUCUGCAGUCAA

>piRNA-L-355_igs

UGGCCAAGGAUGAGAACUCUAAUCUGAUUUU

>piRNA-L-358_igs

GAAGAGCUGACAGCCUAGACUAACGACAU

>piRNA-L-360_igs

GGAAAGAAGACCCUGUUGAGCUUGACUCU

>piRNA-L-361_igs

GCUCAGAAAAUACCUUUCAGUCACACAUU

>piRNA-L-362_igs

AAAGUGAAGACAUGAGAUCCAACUCUGAGC

>piRNA-L-363_igs

GCUCAGAACAAUGCUCUCAUCAGUGAA

>piRNA-L-364_igs

CAGGGUCGGGCCUGGUUAGUACUUGGA

>piRNA-L-365_igs

CUGUCUAGUAAACAGGAGAUCCUGGGU

>piRNA-L-367_igs

AAGCCAGUCAAAUUUAGCAGUGGGGG

>piRNA-L-368_igs

GUAAGUGAAGAUAAAGUGUGUCUGAGG

>piRNA-L-369_igs

CAGAGUGCUCACCAUUACACUAUGGAACC

>piRNA-L-370_igs

UAGCUCAGUCGGUAGAGCAUGAGACU

>piRNA-L-371_igs

CUGGACUGAGGGAAAUAAUCUAUUCUGAGGCU

>piRNA-L-372_igs

UUGGUGGUAUAGUGGUAAGCAUAGCUG

>piRNA-L-373_igs

UCCAAGUACUAACCAGGCCCGACCCUG

>piRNA-L-377_igs

CAGGGUCGGGCCUGGUUAGUACUUGGA

>piRNA-L-378_igs

UUUGGUGUAUGUGCUUGGCUGAGGAGCC

>piRNA-L-381_igs

CAGAGUGCUCACCAUUACACCAUGGAACC

>piRNA-L-382_igs

CAGGAAUCCUAACCGCUAGACCAUAUGGGA

>piRNA-L-383_igs

CUCAGAACCACACAGAGAUUGCAUCACU

>piRNA-L-384_igs

CCUGGGAAUACCGGGUGCUGUAGGCUUU

>piRNA-L-385_igs

UGAGCCGAGAUCACGCCACUGCACUCC

>piRNA-L-386_igs

ACCAGAGUUUCCUCUGGCUUUGCCCU

>piRNA-L-387_igs

GUCCAUGAUGAUUUCAAGUUAUCCCUG

>piRNA-L-389_igs

GUCAAUGAUGUAUUCUUCUUGGAACUGAAU

>piRNA-L-390_igs

CCUCAGACCCCCGGGUGUCAAAGGUCCCGG

>piRNA-L-392_igs

UGACAUUCAGAGCACUGGGCAGAAAU

>piRNA-L-393_igs

CUGAAAUGAAGAGAAUACUCAUUGCUGA

>piRNA-L-394_igs

GCGUUGGUGGUAUAGUGGUGAGCAUAGCUGC

>piRNA-L-399_igs

GUCAGAAUGGUCGAGCGGUCUAAGGC

>piRNA-L-402_igs

UACUGUGAUGAGCUCAGAUGGGGAGACUGAGG

>piRNA-L-404_igs

GUGACACAUGUUUAACGGCCGCGGUAC

>piRNA-L-406_igs

ACUUCCUUUACCUACAUUGUUCCAACAUGCC

>piRNA-L-408_igs

UCUCAGGUAAACAGGUGGGCAUCACAGCU

>piRNA-L-409_igs

GGCUGGUCCGAGUGCAGUGGUGUUUACAACU

>piRNA-L-410_igs

AACAUUCAACGCUGUCGGUGAGUUUG

>piRNA-L-411_igs

CAGUGCUCUAACCCCUGAGCUAUGGAGCC

>piRNA-L-412_igs

UAACCAGGCCCGACCCUGCUUAGCUUCCGAG

>piRNA-L-417_igs

GCCAGAUCAGCCGAAUCAACCCUGGCG

>piRNA-L-418_igs

ACCCAGGAUCUCCUGUUUACUAGACAG

>piRNA-L-420_igs

CGGGUCGGAGUUAGCUCAAGCGGUUAC

>piRNA-L-421_igs

ACCACUCAGACCGCGUUCUCUCCCUC

>piRNA-L-422_igs

CAGAAUGCUAACCAUUACACGAUGGAACC

>piRNA-L-423_igs

GCCCAGCACGCUUCCGCUGCGCCACUCUGCU

>piRNA-L-426_igs

UGGCAACAACACAUCAACAGUAGGGU

>piRNA-L-427_igs

CAGGGUCGGGCCUGGUUAGUACUUGGA

>piRNA-L-428_igs

AGGAGUUCUGGGCUGUAGUGCGCUAUGC

>piRNA-L-429_igs

GUUCAUCCCACAGUGCCAGUUCUGCUUACCAA

>piRNA-L-430_igs

CAUGCGCUCUACCACUGAGCUACAUCCCC

>piRNA-L-432_igs

GGUCUGAACUCAGAUCACGUAGGACUU

>piRNA-L-434_igs

UUCUCACUACUGCACUUGACUAGUCU

>piRNA-L-436_igs

UACAGUCCAAUGCUUCACUCAGCCAUUUUACC

>piRNA-L-439_igs

GUAUUAGAGGCACCGCCUGCCCAGUGAC

>piRNA-L-440_igs

CCAUGGGUGGUUCAGUGGUAGAAUUC

>piRNA-L-442_igs

GGUCCCAUGGUGUAAUGGUUAGCACUCUG

>piRNA-L-443_igs

UACAGUCCAAUGCUUCACUCAGCCAUUUUACC

>piRNA-L-444_igs

UUCUCACUACUGCACUUGACUAGUCU

>piRNA-L-447_igs

GAUGCCUGGGAGUUGCGAUCUGCCCG

>piRNA-L-448_igs

UGAGCCGAGAUCACGCCACUGCACUCC

>piRNA-L-450_igs

CCGAAUCCUAACCAGUAGACUACCAGGGA

>piRNA-L-451_igs

CACAAAAUGUCUGAACCUGCGGUUCCU

>piRNA-L-452_igs

GCCUUAGACUGCUCGGUCAUCCUGAC

>piRNA-L-453_igs

CCGGGGACCUCUUGAUCUGCAGUCAAAU

>piRNA-L-455_igs

ACAACCCCCCACUGCUAAAUUUGACUGGCUU

>piRNA-L-456_igs

GCCCAGCACGCUUCCGCUGCGCCACUCUGCU

>piRNA-L-458_igs

CAGAUGUGAUAACCACUACACUAUGGAACC

>piRNA-L-459_igs

AGACUAGUCAAGUGCAGUAGUGAGAA

>piRNA-L-461_igs

AGACUAGUCAAGUGCAGUAGUGAGAA

>piRNA-L-462_igs

CCUGGACUCAAGCGAUCCUCCAGCCUCAGCCU

>piRNA-L-464_igs

AUCAAUGAUGAAACUAGCCAAAUCUGAGC

>piRNA-L-465_igs

UACUAGAGAAGUUUCUCUGAACGUGUAGAGC

>piRNA-L-466_igs

UUGGCAGUUCAGUGGUAGAAUUCUCGC

>piRNA-L-467_igs

GGCUAAGAUCAAGUGUAGUAUCUGUUC

>piRNA-L-468_igs

CACCAUGAUGGAACUGAGGAUCUGAGGAA

>piRNA-L-469_igs

GUUCAGAAAGGCCAUUUUCAUUCAGCCCC

>piRNA-L-473_igs

UCCCUGGUGGUCUAGUGGCUAGGAUUCGGCA

>piRNA-L-474_igs

GCCUUAGACCACUCGGCCAUCCUGAC

>piRNA-L-475_igs

UUGUAAACACCACUGCACUCGGACCAGCC

>piRNA-L-478_igs

AAAGCCUACAGCACCCGGUAUUCCCAGG

>piRNA-L-479_igs

UGGAAUCCUAACCACUAGACCACCAGGGA

>piRNA-L-480_igs

ACCCCCCACUGCUAAAUUUGACUGGCUUU

>piRNA-L-481_igs

CUGAAUGAUGAUAUCCCACUAACUGAGC

>piRNA-L-482_igs

UGGAGGUGAUGAACUGUCUGAGCCUGACC

>piRNA-L-484_igs

AGGGAGAUGAAGAGGACAGUGACUGAGAGAC

>piRNA-L-485_igs

AGGAGUUCUGGGCUGUAGUGCGCUAUG

>piRNA-L-486_igs

CCGGACAGGGACAGGAUUGACAGAUUGAU

>piRNA-L-487_igs

GCCUUGGUGGUGCAGUGGUAGAAUUCUCGCCU

>piRNA-L-488_igs

AUGGAUAAGGCACUGGCCUCCUAAGCC

>piRNA-L-489_igs

AGGCUGUGAUGGACCUGGCUGAGCCU

>piRNA-L-490_igs

AUCAGAACUUCCACAACAUCAUGUGUU

>piRNA-L-491_igs

UAAGAAUUCUACCACUGAACCACCCAUGC

>piRNA-L-492_igs

UCCAAGUACUAACCAGGCCCGACCCUGCUU

>piRNA-L-495_igs

GGCUUAGGAGGCCAGUGCCUUAUCCAU

>piRNA-L-497_igs

UCCAAGUACUAACCAGGCCCGACCCUGC

>piRNA-L-499_igs

GACCAACUGACCCAUGUUCAACUGCUGU

>piRNA-L-500_igs

AAUCAGAUUUCAGAGUCUCAACAGCAAGU

>piRNA-L-501_igs

AGGAGUUCUGGGCUGUAGUGCGCUAUG

>piRNA-L-502_igs

CUGCCAUGAUGCUAGACUCCUGAGCAGA

>piRNA-L-503_igs

AGGGAGAUGAAGAGGACAGUGACUGAGAGAC

>piRNA-L-504_igs

UCAGAACAUCCGAGAAAAUCAUGUGGU

>piRNA-L-505_igs

GAGUAGAGUGCUUAGUUGAACAGGGCCC

>piRNA-L-506_igs

UGGGAAUACCGGGUGCUGUAGGCUUUU

>piRNA-L-507_igs

UGAAAUGAUGGCAAUCAUCUUUCGGGACU

>piRNA-L-509_igs

CUUUCUGUGUGGAAUUUGAAUAUCUGAAA
